# Surveillance arterioveNous fistulAs using ultRasound (SONAR) trial in haemodialysis patients: a study protocol for a multicentre observational study

**DOI:** 10.1136/bmjopen-2019-031210

**Published:** 2019-07-23

**Authors:** James Richards, Mohammed Hossain, Dominic Summers, Matthew Slater, Matthew Bartlett, Vasilis Kosmoliaptsis, Edward CF Wilson, Regin Lagaac, Anna Sidders, Claire Foley, Emma Laing, Valerie Hopkins, Chloe Fitzpatrick-Creamer, Cara Hudson, Helen Thomas, Sam Turner, Andrew Tambyraja, Subash Somalanka, James Hunter, Sam Dutta, Sarah Lawman, Tracey Salter, Mohammed Aslam, Atul Bagul, Rajesh Sivaprakasam, George Smith, Zia Moinuddin, Simon Knight, Paul Gibbs, Reza Motallebzadeh, Nicholas Barnett, Gavin Pettigrew

**Affiliations:** 1 Department of Surgery, Addenbrooke’s Hospital, Cambridge, UK; 2 Department of Surgery, Cambridge University, Cambridge, UK; 3 Vascular Studies Unit, Addenbrooke’s Hospital, Cambridge, UK; 4 Department of Nephrology and Renal Transplantation, Royal Free London NHS Foundation Trust, London, UK; 5 Cambridge Centre for Health Services Research, University of Cambridge, Cambridge, UK; 6 Cambridge Dialysis Centre, Cambridge University Hospitals, Cambridge; 7 Clinical trials Unit, NHS Blood and Transplant, Cambridge, UK; 8 Renal and Transplant, North Bristol NHS Trust, Westbury on Trym, UK; 9 Department of Clinical Surgery, Royal Infirmary of Edinburgh, Edinburgh, UK; 10 Department of Nephrology, Epsom and St Helier University Hospitals NHS Trust, Epsom, UK; 11 Renal transplantation and nephrology, University Hospitals Coventry and Warwickshire NHS Trust, Coventry, UK; 12 Nottingham Renal and Kidney Transplant Unit, Nottingham University Hospitals NHS Trust, Nottingham, UK; 13 Sussex Kidney Unit, Brighton and Sussex University Hospitals NHS Trust, Brighton, UK; 14 Nephrology Department, Frimley Health NHS Foundation Trust, Frimley, UK; 15 Department of Vascular Surgery, Imperial College London Department of Surgery and Cancer, London, UK; 16 Department of Surgery, University Hospitals of Leicester NHS Trust, Leicester, UK; 17 Department of Surgery, Barts Health NHS Trust, London, UK; 18 Hull York Medical School, Hull University Teaching Hospitals NHS Trust, Hull, UK; 19 Renal Transplant Unit, Manchester University NHS Foundation Trust, Manchester, UK; 20 Nuffield Department of Surgical Sciences, University of Oxford, Oxford, UK; 21 Renal Transplant Department, Portsmouth Hospitals NHS Trust, Portsmouth Hospitals NHS Trust, Portsmouth, UK; 22 Transplant Unit, Guy’s and Saint Thomas' Hospitals NHS Trust, London, UK

**Keywords:** end stage renal failure, dialysis, surgery, ultrasound

## Abstract

**Introduction:**

Arteriovenous fistulas (AVFs) are considered the best and safest modality for providing haemodialysis in patients with end-stage renal disease. Only 20% of UK centres achieve the recommended 80% target for achieving dialysis of the prevalent dialysis population via permanent access (as opposed to a central venous catheter). This is partly due to the relatively poor maturation rate of newly created fistulas, with as many as 50% of fistulas failing to mature.

The Surveillance Of arterioveNous fistulAe using ultRasound study will examine whether a protocolised programme of Doppler ultrasound (US) surveillance can identify, early after creation, potentially correctable problems in those AVFs that subsequently fail to mature.

**Methods and analysis:**

This is a multicentre observational study that will assess newly created AVFs by Doppler US performed at 2, 4, 6 and 10 weeks after creation. The primary outcome measure will be primary fistula patency at week 10. Secondary outcome measures include: successful use of the fistula; clinical suitability for dialysis; creation of new fistula or radiological salvage; fistula thrombosis; secondary fistula patency rate and patient acceptability.

**Ethics and dissemination:**

The study has been approved by the Cambridgeshire and Hertfordshire Research Ethics Committee and by the Health Research Authority (REC 18/EE/0234). The results generated from this work will be published as open access, within 3 years of trial commencement. We will also present our findings at key national/international renal meetings, as well as support volunteers at renal patient groups to disseminate the trial outcome.

**Trial registration number:**

ISRCTN36033877

**Strengths and limitations of this study**

- It is a prospective observational multicentre study that examines whether a programme of Doppler ultrasound (US) surveillance can identify those newly created arteriovenous fistulas (AVFs) that are unlikely to mature.
- The demonstration that US surveillance can successfully identify newly created AVFs that are unlikely to mature will prompt a second-phase prospective randomised study, in which ‘at-risks’ fistulas are randomised to continued observation (control arm) or attempted surgical/radiological salvage.
- The second-phase study will use the same centres and trial expertise as assembled for the initial observational study and would represent the largest vascular access study yet performed, with ~1200 participants.
- The main limitation of this study is that the primary endpoint uses a surrogate marker of fistula patency (based on US flow characteristics) rather than an independent and functionally relevant endpoint (successful use of the fistula for dialysis).

## Introduction

The kidneys provide a number of key functions, including the excretion of excess fluid and harmful toxins. In patients with end-stage renal disease (ESRD), the excessive build-up of toxins and fluid would be fatal if left untreated. Consequently, patients with ESRD require renal replacement therapy in the form of either kidney transplantation or dialysis. About 70% of patients requiring dialysis opt for haemodialysis (equating to about 20 000 patients a year in the UK).^1^

Arteriovenous fistulas (AVFs) are considered the best modality for providing haemodialysis in patients with ESRD, because haemodialysis via a central venous catheter (CVC) is associated with increased incidence of bloodstream infection, hospitalisation and cost.^2–8^ Patient mortality for patients dialysing via a CVC is about 40% higher than for patients dialysing via an AVF.^9^

Only 20% of UK dialysis centres currently achieve the 80% Renal Association target for dialysis of their prevalent population via definitive access (rather than a CVC).^1 10^ The reasons why such a small proportion achieve dialysis via an AVF are multifactorial, but the relatively poor maturation rate, with as many as 50% of fistulas failing to mature, undoubtedly contributes. Although this may reflect suboptimal arterial inflow that is difficult to rectify, stenosis due to venous intimal hyperplasia also contributes and is potentially correctable by either radiological or surgical intervention. If so, the improved assisted primary fistula patency is likely to increase AVF usage substantially, as well as saving money by avoiding the need to create a further AVF or to dialyse via a CVC. Increased AVF usage may result in improved patient survival by avoiding CVC-related complications and by preserving precious venous ‘capital’ for future fistula formation.

The literature relating to ultrasound (US) surveillance of AVFs is conflicting. This may reflect variations in the type of surveillance adopted, the type of fistula under surveillance and the precise US scanning method. US can reliably identify fistulas that have successfully matured,^11 12^ and although the precise ultrasonic characteristics that constitute a mature fistula continue to be debated,^13^ adoption of a surrogate US definition of maturity avoids exclusion of predialysis patients from vascular access trials.

Few studies have attempted to use US to characterise early maturation, immediately after fistula creation, but those that do suggest that successful fistula maturation is associated with rapid increase in fistula blood flow, even by the first day after formation.^14 15^ Fistula vein diameter may also increase rapidly.^15^ Thus, assessment at these earlier time points may be predictive of later patency. In a study of 153 patients, Itoga *et al* performed early duplex US on newly formed fistulas (4–8 weeks after creation).^16^ A flow limiting stenosis was detected in 40% of patients, of whom 81% underwent subsequent radiological intervention. Assisted primary patency of the fistulas in this group (compared with the cohort without detectable US abnormality) was 83% versus 96% at 6 months and 64% versus 89% at 1 year. There was no control cohort (patients who did not undergo routine surveillance), but the assisted patency reported for the entire study population would appear to be better than generally reported following fistula creation. One randomised study has evaluated routine early US surveillance (2, 4 and 8 weeks after fistula creation, 150 patients) and reported a 13.6% fistula failure/non-maturation rate in the surveillance group, compared with 25.4% in the control group in whom US was performed on the basis of a perceived clinical indication.^17^ This difference did not reach statistical significance, but notably, the study was powered for a 20% difference in maturation. Our proposed study is powered for a 10% difference, with a corresponding increase in numbers of enrolled patients.

### Rationale for study

Only 20% of UK dialysis centres manage to meet the Renal Association guidelines to dialyse their prevalent population via an AVF. Numerous factors contribute to this, but the high early failure rate, with as many as 50% of nascent fistulas failing to mature, is undoubtedly a major factor.

At present, the paucity of evidence suggesting surveillance increases rates of dialysis via the fistula alongside the significant cost and required resources leads to significant heterogeneity in practice across the UK and internationally. This study aims to fill this gap in knowledge by determining whether US surveillance of newly formed AVFs can be used to identify failing fistulas.

We aim to demonstrate whether a structured programme of Doppler ultrasound (US) surveillance will accurately predict structural abnormalities (such as venous intimal hyperplasia) that lead to the failure of a newly created fistula to adequately develop and provide effective access for dialysis. We hypothesise that these detected structural abnormalities would be amenable to correction by interventional radiology or surgery, leading to improved assisted fistula patency. This would likely increase AVF usage substantially, as well as potentially save money by avoiding the need to create a further AVF or to dialyse via a CVC. Increased AVF usage may result in improved patient survival by avoiding CVC-related complications and by preserving precious venous ‘capital’ for future fistula formation.

#### When after fistula creation should US surveillance be performed?

Although 1 year unassisted fistula patency (primary) is approximately 55%, about 60% of these failures will occur within the first 3 months after creation,^18 19^ and it is likely that failures in the first year relate to individuals whose fistula had never developed optimally.^20 21^ This study therefore assesses US surveillance of the fistula during its maturation phase, immediately after creation.

We feel that for a trial to demonstrate that US surveillance improves patency rates for newly created fistulas, two conditions must be met:

1. US can effectively distinguish those newly formed fistulas that are unlikely to mature.
2. Salvage interventions performed on those ‘at-risk’ fistulas are effective.

### Study design

These conditions will be addressed in two phases:

Phase I: A prospective observational cohort study to first determine whether US surveillance can reliably predict fistula failure.

Phase II: A prospective randomised trial that examines 1-year fistula patency and compares US-directed fistula salvage against standard clinical assessment (no US surveillance).

This study protocol covers only phase I of the study. We will only move on to phase II if US is found to be effective at identifying ‘at-risk’ AVFs: this will be covered by a separate protocol. The outputs of phases I and II will form the primary inputs into a decision model predicting the incremental cost effectiveness of US surveillance versus standard clinical assessment.

**Figure 1:**
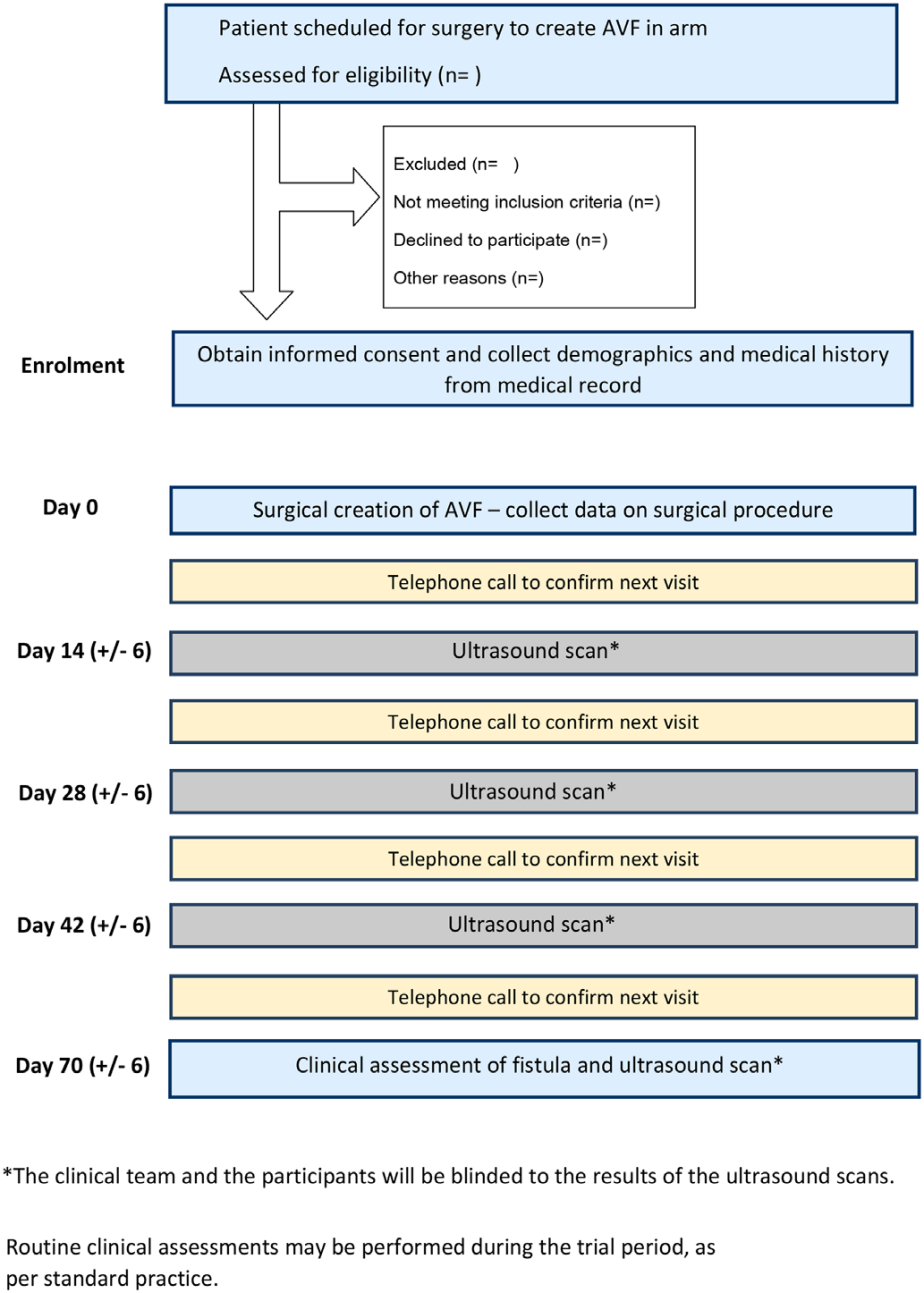
SONAR study flow chart flow chart of patient journey from screening and enrolment through to final study scan and clinical assessment within the SONAR trial. AVF, arteriovenous fistula; SONAR, Surveillance Of arterioveNous fistulAe using ultRasound.

Phase I will recruit patients who are either predialysis or already established on haemodialysis via a central venous catheter (see figure 1). Consenting patients will undergo serial US scanning at weeks 2, 4, 6 and 10 after fistula formation in addition to standard care (such as regular clinical assessment) as per local centre policy. The US findings will be blinded, that is, will not be relayed to the participant or to the participant’s clinical team.

### Potential benefits and risks of study

The only direct benefit to the participating patient is the detection of fistula thrombosis at an earlier juncture than would normally occur with routine clinic follow-up. We anticipate, however, that the incentive to participate in a trial that clarifies the role of US surveillance of newly formed AVFs will enable recruitment rates to be met.

Doppler US is a low risk form of imaging, as it does not use ionising radiation, and as such this observational study poses very low risk to participants. Acoustic power output and duration of exposure to US should not exceed those of a typical diagnostic examination, with exposure kept as low as reasonably achievable.^22^ While no patient injury has been recorded from non-contrast-enhanced US at diagnostic levels, bioeffects including significant heating and cavitation have been demonstrated at higher intensities.

## Methods and analysis

### Study hypothesis

Doppler US surveillance can reliably predict failing nascent AVFs by identifying potentially correctable anatomical defects.

### Intervention

This study will be performed in the UK across 15 participating centres starting 1 August 2018 and with the aim to complete recruitment by 31 October 2019. Consenting participants will be observed for 10 weeks following creation of their AVF and will undergo Doppler US scans during weeks 2, 4, 6 and 10. Routine clinical examination will be undertaken as per local policy with a final clinical examination at week 10 to evaluate the fistula.

### Doppler US surveillance

Duplex Doppler US machines used for the study must have B-mode imaging frequencies of at least 7 MHz. The machines will have both high (>12 MHz), midrange (>7 MHz) and curvilinear (approximately 3 MHz) probes. Scans will be carried out by vascular scientists, surgeons, sonographers and other personnel trained in arteriovenous fistula surveillance by Doppler US; training days have been provided to ensure a standardised approach. The parameters measured for the study will be recorded on an agreed study proforma and include: brachial artery (or subclavian/axillary artery in the presence of a high bifurcation) flow volume and resistance index, outflow vein diameter, anastomotic diameter, AVF depth, presence of a flow limiting stenosis and fistula thrombosis.

### Definitions used within study

#### Primary patency

The interval between access creation to thrombosis event or first surgical/radiological intervention.

#### Secondary patency

The interval between access creation to abandonment of the access including all radiological and surgical salvage procedures in between.

#### Clinical maturation

Suitability to cannulate based on clinical examination.

#### Functional maturation

Ability for the access to achieve adequate dialysis.

### Inclusion criteria

1. Aged 16 years or older.
2. Can provide fully informed consent.
3. Have ESRD and require haemodialysis or are likely to do so imminently.
4. Are due creation of a wrist or elbow AVF, including the following types of fistula with a minimal acceptable threshold of 2 mm venous diameter (with a tourniquet applied) at the site chosen:
  - Radiocephalic.
  - Ulnobasilic.
  - Brachiocephalic.
  - Brachiobasilic.

### Exclusion criteria

1. Attempted formation of proximal neoanastomosis at the forearm cephalic and basilic venous systems following failure of a standard radiocephalic or ulnobasilic fistula.
2. Known central venous stenosis (including those who undergo simultaneous central venous angioplasty/stenting and AVF creation).
3. Anticipated that it will not be possible to perform serial US scanning.

### Primary outcome measure

Primary fistula patency at week 10, according to surrogate US parameters. This is assessed using venous diameter and blood flow measurements:

- Wrist fistula considered to be patent if there is a minimum venous diameter of 4 mm with a blood flow measurement of >400 mL/min.
- Elbow fistula, considered to be patent if there is a minimum venous fistula diameter of 5 mm, with a blood flow measurement of >500 mL/min.

### Secondary outcome measures

1. For those patients established on dialysis, successful use of the fistula, determined by its use for dialysis on 3 separate occasions during the 10 weeks after the AVF surgical creation.
2. Clinical suitability for dialysis based on examination alone according to local practice, assessed 10 weeks after AVF surgical creation.
3. Formation of a new fistula (including fashioning of proximal neoanastomosis) or radiological salvage procedure, measured by collecting the number and type of these interventions during the 10 weeks after AVF surgical creation.
4. Fistula thrombosis rate: the number of fistulae that thrombose during the 10 weeks after surgical creation.
5. Secondary fistula patency rate: measured by the time interval (in days) between AVF creation until abandonment of the AVF, including all radiological and surgical salvage procedures in between, during the 10 weeks after AVF surgical creation.
6. Patient acceptability based on the proportion of patients that complete their study US scans 10 weeks after AVF surgical creation.

### Blinding

Both the patient and the treating clinical team will be blinded to the results of the Doppler US.

The only acceptable reasons for unblinding are:

1. The participating centre’s local standard of care requires a scan, or a clinical need for a scan is identified, in which case the centre will have access to study scan data for that time point (but not the other study scans) to avoid unnecessary additional scans being scheduled, or:
2. During a trial scan, the AVF is seen to be thrombosed, in which case this information would be shared with the clinical care team to enable appropriate care to continue. In such cases, no further study scans would be required. Clinical outcome data will still be collected at week 10.

### Sample size

We have estimated that 20% of fistulas fail early and that early US has a positive predictive value (PPV; number of true positives/number of predicted positives) of 72% for predicting non-maturation. To estimate this with ±10% precision (ie, the 95% CI is from 62% to 82%), 78 predicted failures are required. We estimate that US predicts failure in 25% of fistulas meaning 312 fistulas are required in the study. Allowing for 10% dropout, 347 fistulas will be recruited. To predict primary fistula patency for the phase II trial (not part of this protocol), we anticipate that two models will be required and therefore we will have two PPVs—one for wrist fistulas and one for elbow fistulas. Assuming a ratio of 50:50 for wrist to elbow fistulas, the precision CI will be from 55.3% to 85.2%.

### Analysis plan

All analyses will be performed according to intention to treat. The analysis population will include all participants enrolled into the study, including those whose fistula failed within the first 2 weeks postsurgery and those enrolled in error.

#### Interim monitoring and analyses

The feasibility of recruitment will be assessed based on data between month 3 and month 9 of the recruitment period. Stop–go criteria for expanding to complete the full first phase trial will be used. The trial will be stopped if fewer than 80 patients have been recruited, additional centres will be recruited if fewer than 120 patients have been recruited, otherwise the trial will continue as planned.

#### Analysis of primary and secondary outcomes

The primary fistula patency rate at week 10 will be calculated alongside an exact 95% CI based on all participants enrolled. It will also be calculated based on participants whose fistulas did not fail early. Patency will be assessed using the results from each US scan up to week 10.

The secondary outcomes will be presented using descriptive summary statistics.

Mixed multivariable logistic regression will be used to model primary fistula patency by 10 weeks, which will then be used as a risk-score calculator in the phase II trial. Two models will be built, one for wrist fistulas and one for elbow fistulas. Measurements taken from the first scan performed at week 2 will be considered as parameters in the model initially, and further parameters from either the second scan or the third scan will then be considered. The choice of second or third scan will be based on the best fitting parameters, as assessed by significance level. A random effect for the participant will be included in the models to account for multiple scan data per participant. The aim will be to build parsimonious models, which contain the minimum number of measurements required to effectively predict primary fistula patency. A receiver operating characteristic curve analysis of the developed risk scores will be used to assist decisions regarding an appropriate cut-off for an indicator for when intervention is required for both wrist and elbow fistulas. The PPV will be calculated alongside an exact 95% CI for the chosen risk score cut-off.

It is anticipated that some participants will not attend all scans, and therefore, some scan data will be missing. Levels of missing data will be summarised for each of the scan time points and will be considered when choosing which scan results to use in the final model. Any missing primary and secondary outcome data will not be imputed. Missing data for parameters in the modelling of primary fistula patency will be imputed using multiple imputation if the level of missing data is greater than 10%.

### Data management

Study specific data, which is non-identifiable, will be collected at each site on the case report form (CRF). Each participant will have a unique identification allocated to them which will be recorded on the CRF for reporting purposes. Only study site will have access to the identifiable information to maintain participant confidentiality. CRF data will be submitted to NHS Blood and transplant (NHSBT) Clinical Trials Unit (CTU) at prespecified intervals and logged into the regulatory compliant, secure MACRO database. Only authorised personnel at NHSBT CTU will have password-protected access to the study database.

CRFs, clinical notes and administrative documentation will be kept in a secure location (eg, locked filing cabinets in a room with restricted access) and held for 10 years after the end of the study. During this period, all data will be accessible to the competent authorities and the sponsor with suitable notice.

### Study closure

The study will be closed after the last 10-week appointment of the last recruited patient is completed and all data have been received.

### Patient and public involvement

We discussed trial design and ethical considerations with a number of our haemodialysis patients, and a focus group consisting of 12 lay individuals. In addition to evaluating the trial as a whole, they specifically considered the two-stage design of the trial and the time commitment for participants. They also supported the blinding of results from the patient’s medical team.

### Ethics and dissemination

#### Ethical and regulatory issues

The Cambridgeshire and Hertfordshire Research Ethics Committee has approved this study (18/EE/0234). SONAR complies with the Declaration of Helsinki and will be conducted in compliance with the approved protocol, the principles of Good Clinical Practice, the UK Data Protection Act, the General Data Protection Regulation and the UK Policy Framework for Health and Social Care Research.

### Ethical considerations of SONAR study

As the study is observational with a small likelihood of direct benefit to participants, their involvement will be mainly altruistic. This will be made clear during discussions with potential participants and is clearly stated in the patient information sheet.

### Consent

The rights of the patient to refuse to participate in the study without giving a reason will be respected. After the participant has entered into the study, the clinician remains free to treat the patient according to best standards of care, irrespective of their involvement in the study. The participant will remain within the study for the purposes of follow-up and for data analysis. Similarly, the participant will remain free to change their mind to participate at any time without giving a reason and without prejudicing his/her further treatment.

### Publication and dissemination plan

The results generated from this work will be published in open access, peer-reviewed papers and presented at national/international conferences.

### Access to data

The datasets generated during and/or analysed during the current study will be available on request from Mr Gavin Pettigrew (gjp25@cam.ac.uk). Participants have consented to non-identifiable results to be publicly available. No identifiable data will be shared.

## Discussion

Surveillance Of arterioveNous fistulAe using ultRasound (SONAR) will be one of the largest observational vascular access studies yet performed. If Doppler US accurately identifies anatomically correctable lesions that are preventing maturation (‘salvageable fistula’), this will be used to power and justify a second phase interventional study that addresses whether timely surgical and/or radiological intervention improves fistula patency rates. Conversely, if the SONAR study does not identify a role for routine Doppler US surveillance of newly formed arteriovenous fistulas, this would have immediate relevance for studies in other countries that are similarly using Doppler US to assess fistula maturation.^15 21 23^

## Supplementary Material

Reviewer comments

Author's manuscript
