# Supplementary material for: Surveillance arterioveNous fistulAs using ultRasound (SONAR) trial in haemodialysis patients: a study protocol for a multicentre observational study
Source: BMJ Open. 2019 Jul 23;9(7):e031210. doi: 10.1136/bmjopen-2019-031210 (PMC6661628; doi:10.1136/bmjopen-2019-031210)
Supplement: Author's manuscript [file bmjopen-2019-031210.draft_revisions.pdf]

BMJ Open

BMJ Open is committed to open peer review. As part of this commitment we make the peer review history of every article we publish publicly available.

When an article is published we post the peer reviewers' comments and the authors' responses online. We also post the versions of the paper that were used during peer review. These are the versions that the peer review comments apply to.

The versions of the paper that follow are the versions that were submitted during the peer review process. They are not the versions of record or the final published versions. They should not be cited or distributed as the published version of this manuscript.

BMJ Open is an open access journal and the full, final, typeset and author-corrected version of record of the manuscript is available on our site with no access controls, subscription charges or pay-per-view fees (http://bmjopen.bmj.com).

If you have any questions on BMJ Open's open peer review process please email info.bmjopen@bmj.com

BMJ Open

A study protocol for the Surveillance Of arterioveNous fistulAe using ultRasound (SONAR) trial

Journal:	*BMJ Open*
Manuscript ID	bmjopen-2019-031210
Article Type:	Protocol
Date Submitted by the Author:	22-Apr-2019

Complete List of Authors:	Richards, James; Cambridge University, Department of Surgery; Addenbrooke's Hospital, Department of Surgery<br>Hossain, Mohammed; Addenbrooke's Hospital, Department of Surgery<br>Summers, Dominic ; Cambridge University, Department of Surgery; Addenbrooke's Hospital, Department of Surgery<br>Slater, Matthew; Addenbrooke's Hospital<br>Bartlett, Matthew; Royal Free London NHS Foundation Trust<br>Kosmoliaptsis, Vasilis; Cambridge University, Department of Surgery; Addenbrooke's Hospital, Department of Surgery<br>Wilson, Edward; University of Cambridge, Cambridge Centre for Health Services Research<br>Lagaac, Regin; Addenbrooke's Hospital<br>Sidders, Anna; NHSBT Clinical Trials Unit<br>Foley, Claire; NHSBT Clinical Trials Unit<br>Laing, Emma; NHSBT Clinical Trials Unit<br>Hopkins, Valerie; NHSBT Clinical Trials Unit<br>Fitzpatrick-Creamer, Chloe; NHSBT Clinical Trials Unit<br>Hudson, Cara; NHSBT Clinical Trials Unit<br>Thomas, Helen; NHSBT Clinical Trials Unit<br>Turner, Sam; North Bristol NHS Trust<br>Tambyraja, Andrew; Royal Infirmary of Edinburgh<br>somalanka, Subash; Epsom and St Helier University Hospitals NHS Trust<br>Hunter, James; University Hospitals Coventry and Warwickshire NHS Trust<br>Dutta, Sam; Nottingham University Hospitals NHS Trust<br>Lawman, Sarah; Brighton and Sussex University Hospitals NHS Trust; Epsom and St Helier University Hospitals NHS Trust<br>Salter, Tracey; Frimley Health NHS Foundation Trust<br>Aslam, Mohammed; Imperial College London Department of Surgery and Cancer, Department of Vascular Surgery<br>Bagul, Atul; University Hospitals of Leicester NHS Trust<br>Sivaprakasam, Rajesh; Barts Health NHS Trust<br>Smith, George; Hull University Teaching Hospitals NHS Trust<br>Moinuddin, Zia; Manchester University NHS Foundation Trust<br>Knight, Simon; University of Oxford,<br>Gibbs, Paul; Portsmouth Hospitals NHS Trust, Renal Transplant Department, Portsmouth Hospitals NHS Trust<br>Motallebzadeh, Reza; Royal Free London NHS Foundation Trust<br>Barnett, Nicholas; Guy's and Saint Thomas' Hospitals NHS Trust<br>Pettigrew, Gavin ; Cambridge University, Department of Surgery; Addenbrooke's Hospital, Department of Surgery

Keywords:	End stage renal failure < NEPHROLOGY, Dialysis < NEPHROLOGY, SURGERY, Ultrasound < RADIOLOGY & IMAGING

SCHOLARONE™
Manuscripts

A study protocol for the Surveillance Of arterioveNous fistulAe using ultRasound (SONAR) trial

James Richards ^[1,2], Mohammed Hossain ^[1], Dominic Summers [1,2], Matthew Slater [1], Matthew Bartlett [3], Vasilis Kosmoliaptsis [1,2], Edward Wilson [2], Regin Lagaac [1], Anna Sidders [4], Claire Foley [4], Emma Laing [4], Valerie Hopkins [4], Chloe Fitzpatrick-Creamer [4], Cara Hudson [4], Helen Thomas [4], Sam Turner [5], Andrew Tambyraja [6], Subash Somalanka [7], James Hunter [8], Sam Dutta [9], Sarah Lawman [7,10], Tracey Salter [11], Mohammed Aslam [12], Atul Bagul [13], Rajesh Sivaprakasam [14], George Smith [15], Zia Moinuddin [16], Simon Knight [17], Paul Gibbs [18], Reza Motallebzadeh [3], Nicholas Barnett [19], Gavin Pettigrew *[1,2].

On behalf of the SONAR trial group.

[1] Addenbrooke's Hospital, Hill Road, Cambridge
[2] University of Cambridge, Hill Road, Cambridge
[3] Royal Free London NHS Foundation Trust
[4] NHS Blood and Transplant Clinical Trials Unit
[5] North Bristol NHS Trust
[6] Royal Infirmary of Edinburgh, NHS Lothian
[7] Epsom and St Helier University Hospitals NHS Trust
[8] University Hospital Coventry and Warwickshire NHS Trust
[9] Nottingham University Hospitals NHS Trust
[10] Brighton & Sussex University Hospitals NHS Trust
[11] Frimley Health NHS Foundation Trust
[12] Imperial College Healthcare NHS Trust
[13] University Hospitals of Leicester NHS Trust
[14] Bart's Health NHS Trust
[15] Hull University Teaching Hospitals NHS Trust
[16] Manchester University NHS Foundation Trust
[17] Oxford University Hospitals NHS Foundation Trust
[18] Portsmouth Hospitals NHS Trust)
[19] Guy's & St Thomas' NHS Foundation Trust

* Corresponding author & Chief Investigator: Mr Gavin Pettigrew, Department of Surgery, Box 202, Level E9, Addenbrooke's Hospital, Cambridge Biomedical Campus, Hills Road, Cambridge, CB2 0QQ, UK. Email: gjp25@cam.ac.uk. Tel: 01223 336976. Fax 01223 762523.

^ Joint first authors

For peer review only

Abstract

Introduction

Arteriovenous fistulas (AVFs) are considered the best and safest modality for providing haemodialysis in patients with End-Stage Renal Disease. Only 20% of UK centres achieve the recommended 80% target for achieving dialysis of the prevalent dialysis population via permanent access (as opposed to a central venous catheter). This is partly due to the

relatively poor maturation rate of newly created fistulas, with as many as 50% of fistulas failing to mature.

The SONAR study will examine whether a protocolised programme of Doppler ultrasound (US) surveillance can identify, early after creation, potentially correctable problems in those AVFs that subsequently fail to mature.

Methods and Analysis
This is a multi-centre observational study that will assess newly created AVFs by Doppler US performed at 2, 4, 6 and 10 weeks after creation. The primary outcome measure will be primary fistula patency at week 10. Secondary outcome measures include: successful use of the fistula; clinical suitability for dialysis; creation of new fistula or radiological salvage; fistula thrombosis; secondary fistula patency rate; and patient acceptability.

Ethics and dissemination
The study has been approved by the Cambridgeshire and Hertfordshire Research Ethics Committee and by the Health Research Authority (REC 18/EE/0234). The results generated from this work will be published as open access, within three years of trial commencement. We will also present our findings at key national / international renal meetings, as well as support volunteers at renal patient groups to disseminate the trial outcome.

Trial Registration number
This study is registered with the ISRCTN Clinical Trials Register (ISRCTN36033877).

Manuscript Word Count: 3615

For peer review only

Strengths and limitations of this study

- It is a prospective observational multi-centre study that examines whether a programme of Doppler Ultrasound (US) surveillance can identify those newly created arteriovenous fistulas (AVFs) that are unlikely to mature.
- The demonstration that US surveillance can successfully identify newly-created AVFs that are unlikely to mature will prompt a second-phase prospective randomised study, in which 'at-risks' fistulas are randomised to continued observation (control arm) or attempted surgical / radiological salvage.
- The second-phase study will use the same centres and trial expertise as assembled for the initial observational study and would represent the largest vascular access study yet performed, with ~1200 participants.
- The main limitation of this study is that the primary end-point uses a surrogate marker of fistula patency (based on US flow characteristics), rather than an independent and functionally-relevant endpoint (successful use of the fistula for dialysis).

Keywords
Arteriovenous Fistula
Vascular Access Surgery
Doppler Ultrasound
Haemodialysis
End-stage Kidney Disease

For peer review only

Introduction

The kidneys provide a number of key functions, including the excretion of excess fluid and harmful toxins. In patients with end-stage renal disease (ESRD), the excessive build-up of toxins and fluid would be fatal if left untreated. Consequently, patients with ESRD require renal replacement therapy (RRT), in the form of either kidney transplantation or dialysis. About 70% of patients requiring dialysis opt for haemodialysis (equating to about 20,000 patients a year in the UK) [1].

Arteriovenous fistulas (AVFs) are considered the best modality for providing haemodialysis in patients with ESRD, because haemodialysis via a central venous catheter (CVC) is associated with increased incidence of bloodstream infection, hospitalisation and cost [2-8]. Patient mortality for patients dialysing via a CVC is about 40% higher than for patients dialysing via an AVF [9].

Only 20% of UK dialysis centres currently achieve the 80% Renal Association target for dialysis of their prevalent population via definitive access (rather than a CVC) [1 10]. The reasons why such a small proportion achieve dialysis via an AVF are multifactorial, but the relatively poor maturation rate, with as many as 50% of fistulas failing to mature, undoubtedly contributes. Although this may reflect sub-optimal arterial inflow that is difficult to rectify, stenosis due to venous intimal hyperplasia also contributes, and is potentially correctable by either radiological or surgical intervention. If so, the improved assisted primary fistula patency is likely to increase AVF usage substantially, as well as saving money by avoiding the need to create a further AVF or to dialyse via a CVC. Increased AVF usage may result in improved patient survival by avoiding CVC-related complications, and by preserving precious venous 'capital' for future fistula formation.

The literature relating to ultrasound (US) surveillance of AVFs is conflicting. This may reflect variations in the type of surveillance adopted, the type of fistula under surveillance and the precise US scanning method. US can reliably identify fistulas that have successfully matured [11 12], and although the precise ultrasonic characteristics that constitute a mature fistula continue to be debated [13], adoption of a surrogate US definition of maturity avoids exclusion of pre-dialysis patients from vascular access trials.

Few studies have attempted to use ultrasound to characterise early maturation, immediately after fistula creation, but those that do suggest that successful fistula maturation is associated with rapid increase in fistula blood flow, even by the first day after formation [14 15]. Fistula vein diameter may also increase rapidly [15]. Thus, assessment at these earlier time points may be predictive of later patency. In a study of 153 patients, Itoga et al. performed early duplex US on newly-formed fistulas (4-8 weeks after creation) [16]. A flow limiting stenosis was detected in 40% of patients, of whom 81% underwent subsequent radiological intervention. Assisted primary patency of the fistulas in this group (compared to the cohort without detectable US abnormality) was 83% vs 96% at six months and 64% vs 89% at one year. There was no control cohort (patients who did not undergo routine surveillance), but the assisted patency reported for the entire study population would appear to be better than generally reported following fistula creation. One randomised study has evaluated routine early US surveillance (2, 4 and 8 weeks after fistula creation,

For peer review only

150 patients), and reported a 13.6% fistula failure/non-maturation rate in the surveillance group, compared to 25.4% in the control group in whom US was performed on the basis of a perceived clinical indication [17]. This difference did not reach statistical significance, but notably, the study was powered for a 20% difference in maturation. Our proposed study is powered for a 10% difference, with a corresponding increase in numbers of enrolled patients.

Rationale for Study
Only 20% of UK dialysis centres manage to meet the Renal Association guidelines to dialyse their prevalent population via an AVF. Numerous factors contribute to this, but the high early failure rate, with as many as 50% of nascent fistulas failing to mature, is undoubtedly a major factor.

At present, the paucity of evidence suggesting surveillance increases rates of dialysis via the fistula alongside the significant cost and required resources, leads to significant heterogeneity in practice across the UK and internationally. This study aims to fill this gap in knowledge by determining whether US surveillance of newly-formed AVFs can be used to identify failing fistulas.

We aim to demonstrate whether a structured programme of Doppler US surveillance will accurately predict structural abnormalities (such as venous intimal hyperplasia) that lead to failure of a newly-created fistula to adequately develop and provide effective access for dialysis. We hypothesise that these detected structural abnormalities would be amenable to correction by interventional radiology or surgery, leading to improved assisted fistula patency. This would likely increase AVF usage substantially, as well as potentially save money by avoiding the need to create a further AVF or to dialyse via a CVC. Increased AVF usage may result in improved patient survival by avoiding CVC-related complications, and by preserving precious venous 'capital' for future fistula formation.

When after fistula creation should US surveillance be performed?
Although one year unassisted fistula patency (primary) is approximately 55%, about 60% of these failures will occur within the first three months after creation [18 19], and it is likely that failures in the first year relate to individuals whose fistula had never developed optimally [20 21]. This study therefore assesses US surveillance of the fistula during its maturation phase, immediately after creation.

We feel that for a trial to demonstrate that US surveillance improves patency rates for newly created fistulas, two conditions must be met:

1. Ultrasound can effectively distinguish those newly-formed fistulas that are unlikely to mature.
2. Salvage interventions performed on those 'at-risk' fistulas are effective.

Study Design
These conditions will be addressed in two phases:
Phase 1: A prospective observational cohort study to firstly determine whether US surveillance can reliably predict fistula failure.

For peer review only

Phase 2: A prospective randomised trial that examines one-year fistula patency and compares US-directed fistula salvage against standard clinical assessment (no ultrasound surveillance).

This study protocol covers only phase 1 of the study. We will only move on to phase 2 if US is found to be effective at identifying 'at-risk' AVFs: this will be covered by a separate protocol. The outputs of phases 1 and 2 will form the primary inputs into a decision model predicting the incremental cost effectiveness of US surveillance versus standard clinical assessment.

Phase 1 will recruit patients who are either pre-dialysis or already established on haemodialysis via a central venous catheter (see Figure 1). Consenting patients will undergo serial US scanning at weeks 2, 4, 6 and 10 after fistula formation in addition to standard care (such as regular clinical assessment) as per local centre policy. The US findings will be blinded, i.e. will not be relayed to the participant or to the participant's clinical team.

Potential benefits and risks of study
The only direct benefit to the participating patient is the detection of fistula thrombosis at an earlier juncture than would normally occur with routine clinic follow up. We anticipate, however, that the incentive to participate in a trial that clarifies the role of ultrasound surveillance of newly-formed AVFs will enable recruitment rates to be met.

Doppler US is a low risk form of imaging, as it does not use ionizing radiation and as such this observational study poses very low risk to participants. Acoustic power output and duration of exposure to ultrasound should not exceed those of a typical diagnostic examination, with exposure kept as low as reasonably achievable [22]. While no patient injury has been recorded from non-contrast enhanced US at diagnostic levels, bioeffects including significant heating and cavitation have been demonstrated at higher intensities.

Methods and Analysis
Study Hypothesis
Doppler US surveillance can reliably predict failing nascent AV fistulas by identifying potentially correctable anatomical defects.

Intervention
This study will be performed in the UK across 15 participating centres. Consenting participants will be observed for 10 weeks following creation of their AV fistula and will undergo Doppler US scans during weeks 2, 4, 6 and 10. Routine clinical examination will be undertaken as per local policy with a final clinical examination at week 10 to evaluate the fistula.

Doppler Ultrasound Surveillance
Duplex Doppler US machines used for the study must have B-mode imaging frequencies of at least 7MHz. The machines will have both high (>12MHz), midrange (>7MHz) and curvilinear (approximately 3MHz) probes. Scans will be carried out by vascular scientists, surgeons, sonographers and other personnel trained in arteriovenous fistula surveillance by Doppler US; training days have been provided to ensure a standardised approach. The

For peer review only - http://bmjopen.bmj.com/site/about/guidelines.xhtml

parameters measured for the study will be recorded on an agreed study proforma and include: brachial artery (or subclavian/axillary artery in the presence of a high bifurcation) flow volume and resistance index, outflow vein diameter, anastomotic diameter, AVF depth, presence of a flow limiting stenosis and fistula thrombosis.

Definitions used within study

Primary patency:
The interval between access creation to thrombosis event or first surgical/radiological intervention.

Secondary patency
The interval between access creation to abandonment of the access including all radiological and surgical salvage procedures in between.

Clinical maturation
Suitability to cannulate based on clinical examination

Functional maturation
Ability for the access to achieve adequate dialysis

Inclusion Criteria

1. Aged 16 years or older
2. Can provide fully informed consent
3. Have end-stage renal disease and require haemodialysis or are likely to do so imminently
4. Are due creation of a wrist or elbow arteriovenous fistula, including the following types of fistula with a minimal acceptable threshold of 2 mm venous diameter (with a tourniquet applied) at the site chosen:
    - Radiocephalic
    - Ulnobasilic
    - Brachiocephalic
    - Brachiobasilic

Exclusion criteria

1. Attempted formation of proximal neo-anastomosis at the forearm cephalic and basilic venous systems following failure of a standard radiocephalic or ulnobasilic fistula.
2. Known central venous stenosis (including those who undergo simultaneous central venous angioplasty / stenting and arteriovenous fistula creation).
3. Anticipated that it will not be possible to perform serial ultrasound scanning.

For peer review only

Primary outcome measure
Primary fistula patency at week 10, according to surrogate ultrasound parameters. This is assessed using venous diameter and blood flow measurements:

- Wrist fistula considered to be patent if there is a minimum venous diameter of 4 mm with a blood flow measurement of >400 mls/min
- Elbow fistula, considered to be patent if there is a minimum venous fistula diameter of 5 mm, with a blood flow measurement of >500 mls/min

Secondary outcome measures
1. For those patients established on dialysis, successful use of the fistula, determined by its use for dialysis on 3 separate occasions during the 10 weeks after the arteriovenous fistula (AVF) surgical creation.
2. Clinical suitability for dialysis based on examination alone according to local practice, assessed 10 weeks after AVF surgical creation.
3. Formation of a new fistula (including fashioning of proximal neoanastomosis) or radiological salvage procedure, measured by collecting the number and type of these interventions during the 10 weeks after AVF surgical creation.
4. Fistula thrombosis rate: the number of fistulae that thrombose during the 10 weeks after surgical creation.
5. Secondary fistula patency rate: measured by the time interval (in days) between AVF creation until abandonment of the AVF, including all radiological and surgical salvage procedures in between, during the 10 weeks after AVF surgical creation.
6. Patient acceptability based on the proportion of patients that complete their study ultrasound scans 10 weeks after AVF surgical creation.

Blinding
Both the patient and the treating clinical team will be blinded to the results of the Doppler US.

The only acceptable reasons for unblinding are:

1. The participating centre's local standard of care requires a scan, or a clinical need for a scan is identified. In which case the centre will have access to study scan data for that timepoint (but not the other study scans) to avoid unnecessary additional scans being scheduled.

Or:

2. During a trial scan, the AVF is seen to be thrombosed, in which case this information would be shared with the clinical care team to enable appropriate care to continue. In such cases, no further study scans would be required. Clinical outcome data will still be collected at week 10.

Sample size
We have estimated that 20% of fistulas fail early and that early ultrasound has a positive predictive value (number of true positives/number of predicted positives, PPV) of 72% for predicting non-maturation. To estimate this with ±10% precision (i.e. the 95% confidence interval is from 62% to 82%), 78 predicted failures are required. We estimate that US

For peer review only

predicts failure in 25% of fistulas meaning 312 fistulas are required in the study. Allowing for 10% dropout, 347 fistulas will be recruited. In order to predict primary fistula patency for the phase 2 trial (not part of this protocol), we anticipate that two models will be required and therefore we will have two PPVs- one for wrist fistulas and one for elbow fistulas. Assuming a ratio of 50:50 for wrist to elbow fistulas, the precision confidence interval will be from 55.3% to 85.2%.

Analysis Plan

All analyses will be performed according to intention to treat. The analysis population will include all participants enrolled into the study, including those whose fistula failed within the first two weeks post-surgery and those enrolled in error.

Interim Monitoring and Analyses

The feasibility of recruitment will be assessed based on data between month 3 and month 9 of the recruitment period. Stop-go criteria for expanding to complete the full first phase trial will be used. The trial will be stopped if fewer than 80 patients have been recruited, additional centres will be recruited if less than 120 patients have been recruited, otherwise the trial will continue as planned.

Analysis of primary and secondary outcomes

The primary fistula patency rate at week 10 will be calculated alongside an exact 95% confidence interval based on all participants enrolled. It will also be calculated based on participants whose fistulas did not fail early. Patency will be assessed using the results from each ultrasound scan up to week 10.

The secondary outcomes will be presented using descriptive summary statistics.

Mixed multivariable logistic regression will be used to model primary fistula patency by 10 weeks, which will then be used as a risk-score calculator in the phase 2 trial. Two models will be built, one for wrist fistulas and one for elbow fistulas. Measurements taken from the first scan performed at week 2 will be considered as parameters in the model initially and further parameters from either the second scan or the third scan will then be considered. The choice of second or third scan will be based on the best fitting parameters, as assessed by significance level. A random effect for the participant will be included in the models to account for multiple scan data per participant. The aim will be to build parsimonious models, which contain the minimum number of measurements required to effectively predict primary fistula patency. A receiver operating characteristic (ROC) curve analysis of the developed risk scores will be used to assist decisions regarding an appropriate cut-off for an indicator for when intervention is required for both wrist and elbow fistulas. The PPV will be calculated alongside an exact 95% confidence interval for the chosen risk-score cut-off.

It is anticipated that some participants will not attend all scans and therefore some scan data will be missing. Levels of missing data will be summarised for each of the scan time points and will be considered when choosing which scan results to use in the final model. Any missing primary and secondary outcome data will not be imputed. Missing data for

For peer review only

parameters in the modelling of primary fistula patency will be imputed using multiple imputation if the level of missing data is greater than 10%.

Data management

Study specific data, which is non-identifiable, will be collected at each site on the Case Report Form (CRF). Each participant will have a unique ID allocated to them which will be recorded on the CRF for reporting purposes. Only study sites will have access to the identifiable information to maintain participant confidentiality. CRF data will be submitted to NHSBT Clinical Trials Unit (CTU) at pre-specified intervals and logged into the regulatory compliant, secure MACRO™ database. Only authorised personnel at NHSBT CTU will have password protected access to the study database.

CRFs, clinical notes and administrative documentation will be kept in a secure location (e.g. locked filing cabinets in a room with restricted access) and held for 10 years after the end of the study. During this period, all data will be accessible to the competent authorities and the Sponsor with suitable notice.

Study Closure

The study will be closed after the last 10-week appointment of the last recruited patient is completed and all data have been received.

Patient and Public Involvement

We discussed trial design and ethical considerations with a number of our haemodialysis patients, as well as focus group consisting of 12 lay individuals. As well as considering the trial as a whole, they specifically considered two-stage design of the trial, the time commitment for participants and also supported the blinding of results from the patient's medical team.

Ethics and dissemination

Ethical and Regulatory Issues

The Cambridgeshire and Hertfordshire Research Ethics Committee has approved this study (18/EE/0234). SONAR complies with the Declaration of Helsinki and will be conducted in compliance with the approved protocol, the principles of Good Clinical Practice (GCP), the UK Data Protection Act, the General Data Protection Regulation and the UK Policy Framework for Health and Social Care Research.

Ethical Considerations of SONAR study

As the study is observational with a small likelihood of direct benefit to participants, their involvement will be mainly altruistic. This will be made clear during discussions with potential participants and is clearly stated in the patient information sheet

Consent

The rights of the patient to refuse to participate in the study without giving a reason will be respected. After the participant has entered into the study, the clinician remains free to treat the patient according to best standards of care, irrespective of their involvement in the study. The participant will remain within the study for the purposes of follow up and for

For peer review only

data analysis. Similarly, the participant will remain free to change their mind to participate at any time without giving a reason and without prejudicing his/her further treatment.

Publication and dissemination plan
The results generated from this work will be published in open access, peer reviewed papers and presented at national/international conferences.

Access to data
The datasets generated during and/or analysed during the current study will be available upon request from Mr Gavin Pettigrew (gjp25@cam.ac.uk). Participants have consented to non-identifiable results to be publicly available. No identifiable data will be shared.

Discussion

SONAR will be one of the largest observational vascular access studies yet performed. If Doppler US accurately identifies anatomically correctable lesions that are preventing maturation ("salvageable fistula"), this will be used to power and justify a second phase interventional study that addresses whether timely surgical and/or radiological intervention improves fistula patency rates. Conversely, if the SONAR study does not identify a role for routine Doppler US surveillance of newly-formed arteriovenous fistulas, this would have immediate relevance for studies in other countries that are similarly using Doppler US to assess fistula maturation [21 23 24].

Authors' contributions

JR, MH, MS, DS, MB, VK, EW, RL, AS, CF, EL, VH, CFC, CH, HT, ST, AT, SS, JH, SD, SL, TS, MA, AB, RS, GS, ZM, SK, PG, RM, NB & GJP contributed to the development and implementation of this protocol and have approved this manuscript. All members of the Trial Group were involved in the development of the study.

SONAR TRIAL Group
Anna Sidders, Cara Hudson, Claire Foley, Valerie Hopkins, Emma Laing, Chloe Fitzpatrick-Creamer, Helen Thomas, Alison Deary (NHS Blood and Transplant Clinical Trials Unit)

Gavin Pettigrew, James Richards, Mohammed Hossain, Dominic Summers, Matthew Slater, Regin Lagaac, Veena Surendrakumar, Tobi Ayorinde, Igor Chipurovski, Subhankar Paul (Cambridge University Hospital)

Simon Knight, Klaus Bond, Elizabeth Hardy, Joanne Widdup, Rachael Potter, Elisabeth Pugh, Karen Parsons, Kathryn Lafferty (Oxford University Hospitals NHS Foundation Trust)

Rajesh Sivaprakasam, Kate Crawford, Amy Bolsworth, Naavalah Ngwa-Ndifor (Bart's Health NHS Trust)

Reza Motallebzadeh, Matthew Bartlett, Rani Badhan (Royal Free London NHS Foundation Trust)

Zia Moinuddin, Helena Edlin, Anna Jerram, Jessica Lai, Joyce Banda, Janet Bendle (Manchester University NHS Foundation Trust)

For peer review only

Sam Turner, Maria Morgan, William Owen, Sue Dawson, Simon Daniel, Karen Allsop, (North Bristol NHS Trust)

Andrew Tambyraja, Sarah-Jane Carmichael, Tom Eadie, Rona Lochiel, Midel Lena (Royal Infirmary of Edinburgh, NHS Lothian)

Nicholas Barnett, Soundrie Padayachee, Philip Eldridge, May Rabuya (Guy's & St Thomas' NHS Foundation Trust)

Subash Somalanka, Jashree Patel, Abbas Ghazanfar, Judy van Selm, Caroline Bodneck, Martia Augustin, Kwame Ansu, Nalin Khosla, Kashif Burney, Karen Dear (Epsom and St Helier University Hospitals NHS Trust)

James Hunter, Carl Tiivas, Samantha Hyndman, Maria Truslove (University Hospital Coventry and Warwickshire NHS Trust)

Sam Dutta, Andrew Beech, Sarah Brand (Nottingham University Hospitals NHS Trust)

Sarah Lawman, Darren Cheal, Mel Smith, Kate Trivedi (Brighton & Sussex University Hospitals NHS Trust)

Tracey Salter, Adnan Bajwa, John Kerr, Ana Fleet, Lianne Chapman, Sarah Gee, Thanuja Weerasinghe (Frimley Health NHS Foundation Trust)

George Smith, Paris Cai, Judith Long, Tracey Rowe (Hull University Teaching Hospitals NHS Trust)

Mohammed Aslam, Jeremy Crane (Imperial College Healthcare NHS Trust)

Atul Bagul, Mary Quashie-Akponeware (University Hospitals of Leicester NHS Trust)

Paul Gibbs (Portsmouth Hospitals NHS Trust)

Funding Statement

This study is funded by National Institute for Health Research (NIHR) Health Technology Assessment Board (grant reference 17/27/11). The views expressed are those of the author(s) and not necessarily those of the NIHR or the Department of Health and Social Care. JR and DS are funded by NIHR Academic Clinical Lectureships.

Competing interests

None declared

For peer review only

References

1. Caskey F, Cullen R. UK Renal Registry 18th Annual Report: Introduction. *Nephron* 2016;132 Suppl 1:1-8. doi: 10.1159/000444814 [published Online First: 2016/04/19]
2. Taylor G, Gravel D, Johnston L, et al. Incidence of bloodstream infection in multicenter inception cohorts of hemodialysis patients. *Am J Infect Control* 2004;32(3):155-60. doi: 10.1016/j.ajic.2003.05.007 [published Online First: 2004/05/22]
3. D'Agata EM, Mount DB, Thayer V, et al. Hospital-acquired infections among chronic hemodialysis patients. *Am J Kidney Dis* 2000;35(6):1083-8. [published Online First: 2000/06/10]
4. Marr KA, Kong L, Fowler VG, et al. Incidence and outcome of Staphylococcus aureus bacteremia in hemodialysis patients. *Kidney Int* 1998;54(5):1684-9. doi: 10.1046/j.1523-1755.1998.00134.x [published Online First: 1998/12/09]
5. Rodriguez-Aranda A, Alcazar JM, Sanz F, et al. Endoluminal colonization as a risk factor for coagulase-negative staphylococcal catheter-related bloodstream infections in haemodialysis patients. *Nephrol Dial Transplant* 2011;26(3):948-55. doi: 10.1093/ndt/gfq481 [published Online First: 2010/08/13]
6. Rosenbaum D, MacRae JM, Djurdjev O, et al. Surveillance cultures of tunneled cuffed catheter exit sites in chronic hemodialysis patients are of no benefit. *Hemodial Int* 2006;10(4):365-70. doi: 10.1111/j.1542-4758.2006.00131.x [published Online First: 2006/10/04]
7. McCann M, Moore ZE. Interventions for preventing infectious complications in haemodialysis patients with central venous catheters. *Cochrane Database Syst Rev* 2010(1):Cd006894. doi: 10.1002/14651858.CD006894.pub2 [published Online First: 2010/01/22]
8. Engemann JJ, Friedman JY, Reed SD, et al. Clinical outcomes and costs due to Staphylococcus aureus bacteremia among patients receiving long-term hemodialysis. *Infect Control Hosp Epidemiol* 2005;26(6):534-9. doi: 10.1086/502580 [published Online First: 2005/07/16]
9. Astor BC, Eustace JA, Powe NR, et al. Type of vascular access and survival among incident hemodialysis patients: the Choices for Healthy Outcomes in Caring for ESRD (CHOICE) Study. *J Am Soc Nephrol* 2005;16(5):1449-55. doi: 10.1681/asn.2004090748 [published Online First: 2005/03/25]
10. Association TR. Vascular Access Guidelines https://renal.org/wp-content/uploads/2017/06/vascular-access.pdf 2015 [2.
11. Ives CL, Akoh JA, George J, et al. Pre-operative vessel mapping and early post-operative surveillance duplex scanning of arteriovenous fistulae. *J Vasc Access* 2009;10(1):37-42.
12. Hindle P, Ritchie J, Griffiths G, et al. Use of early scanning in fistula formation for vascular access. *The Journal of Vascular Access* 2011;12(2):127-29. doi: 10.5301/jva.2010.6072
13. Beathard GA, Lok CE, Glickman MH, et al. Definitions and End Points for Interventional Studies for Arteriovenous Dialysis Access. *Clin J Am Soc Nephrol* 2018;13(3):501-12. doi: 10.2215/cjn.11531116 [published Online First: 2017/07/22]
14. Saucy F, Haesler E, Haller C, et al. Is intra-operative blood flow predictive for early failure of radiocephalic arteriovenous fistula? *Nephrol Dial Transplant* 2010;25(3):862-7. doi: 10.1093/ndt/gfp577

For peer review only

15. Robbin ML, Greene T, Cheung AK, et al. Arteriovenous Fistula Development in the First 6 Weeks after Creation. *Radiology* 2016;279(2):620-9. doi: 10.1148/radiol.2015150385
16. Itoga NK, Ullery BW, Tran K, et al. Use of a proactive duplex ultrasound protocol for hemodialysis access. *J Vasc Surg* 2016;64(4):1042-49 e1. doi: 10.1016/j.jvs.2016.03.442
17. Han A, Min SK, Kim MS, et al. A Prospective, Randomized Trial of Routine Duplex Ultrasound Surveillance on Arteriovenous Fistula Maturation. *Clin J Am Soc Nephrol* 2016;11(10):1817-24. doi: 10.2215/CJN.00620116
18. Wells AC, Fernando B, Butler A, et al. Selective use of ultrasonographic vascular mapping in the assessment of patients before haemodialysis access surgery. *Br J Surg* 2005;92(11):1439-43. doi: 10.1002/bjs.5151 [published Online First: 2005/09/28]
19. Huijbregts HJ, Bots ML, Wittens CH, et al. Hemodialysis arteriovenous fistula patency revisited: results of a prospective, multicenter initiative. *Clin J Am Soc Nephrol* 2008;3(3):714-9. doi: 10.2215/CJN.02950707
20. Robbin ML, Chamberlain NE, Lockhart ME, et al. Hemodialysis arteriovenous fistula maturity: US evaluation. *Radiology* 2002;225(1):59-64. doi: 10.1148/radiol.2251011367 [published Online First: 2002/10/02]
21. Singh P, Robbin ML, Lockhart ME, et al. Clinically immature arteriovenous hemodialysis fistulas: effect of US on salvage. *Radiology* 2008;246(1):299-305. doi: 10.1148/radiol.2463061942 [published Online First: 2007/11/10]
22. American Institute of Ultrasound in Medicine Official Statement on Safety in Training and Research, 2012. 2012
23. Robbin ML, Greene T, Allon M, et al. Prediction of Arteriovenous Fistula Clinical Maturation from Postoperative Ultrasound Measurements: Findings from the Hemodialysis Fistula Maturation Study. *J Am Soc Nephrol* 2018;29(11):2735-44. doi: 10.1681/asn.2017111225 [published Online First: 2018/10/13]
24. Robbin ML, Greene T, Cheung AK, et al. Arteriovenous Fistula Development in the First 6 Weeks after Creation. *Radiology* 2016;279(2):620-9. doi: 10.1148/radiol.2015150385 [published Online First: 2015/12/24]

For peer review only

Figure 1: SONAR study flow chart

SONAR - Surveillance of arteriovenous fistulae using ultrasound (NIHR HTA 17/27/11)

STUDY FLOWCHART

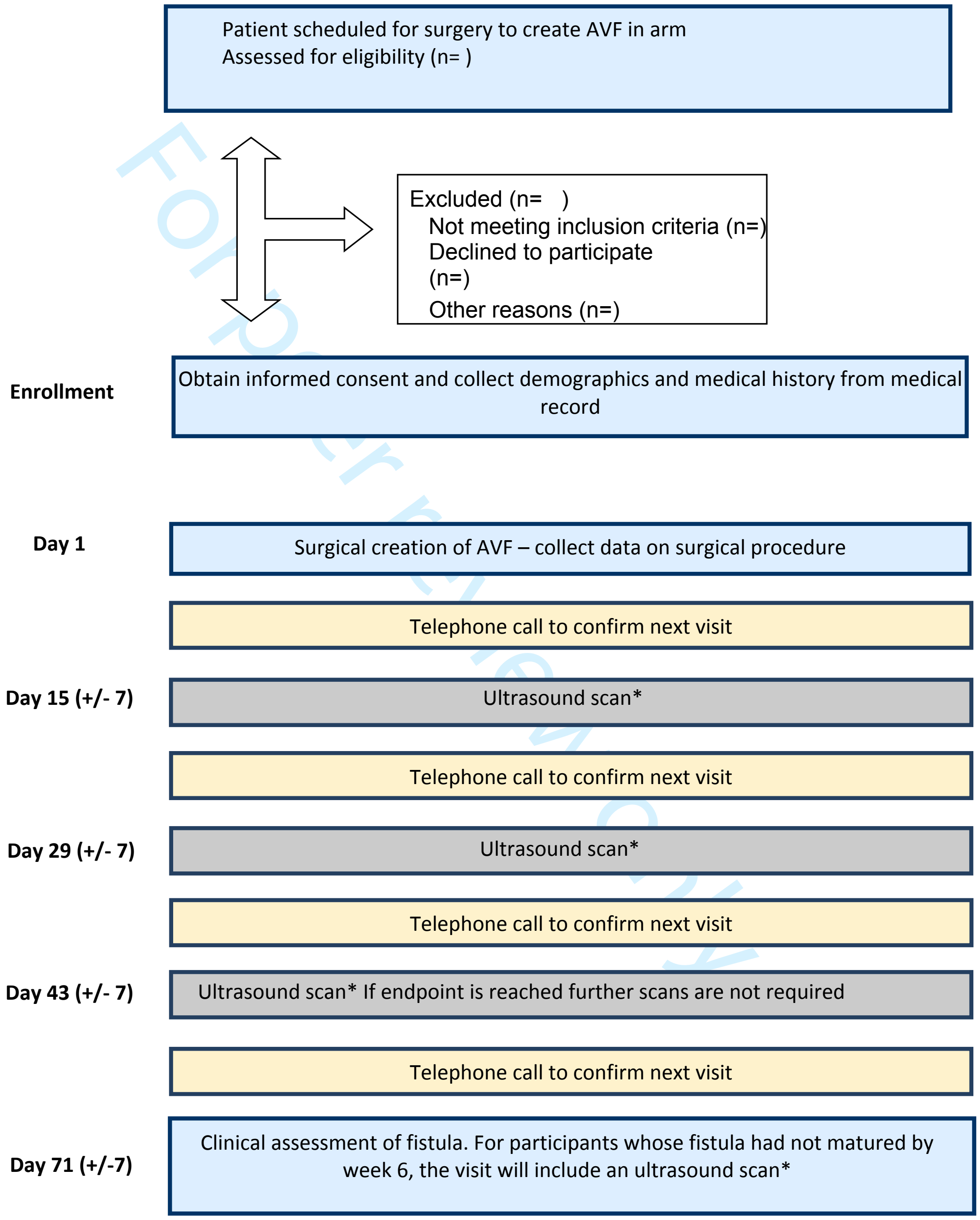

*The clinical team and the participants will be blinded to the results of the ultrasound scans

Routine clinical assessments may be performed during the trial period, as per standard practice.

For peer review only

SONAR - Surveillance of arteriovenous fistulae using ultrasound (NIHR HTA 17/27/11)

STUDY FLOWCHART

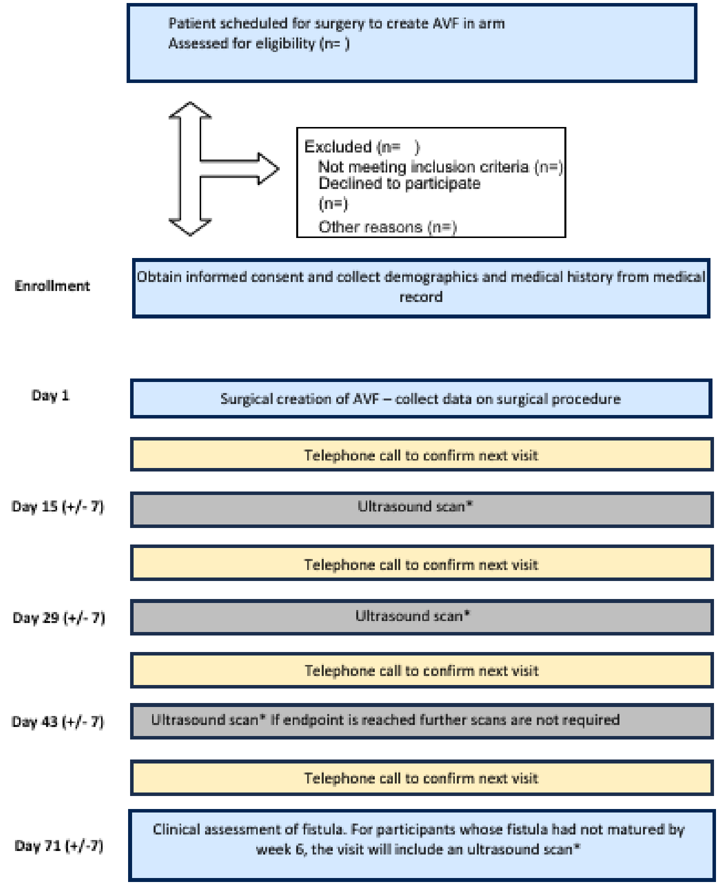

*The clinical team and the participants will be blinded to the results of the ultrasound scans

Routine clinical assessments may be performed during the trial period, as per standard practice.

BMJ Open

A study protocol for the multi-centre observational study: Surveillance Of arterioveNous fistulAe using ultRasound (SONAR) trial in haemodialysis patients

Journal:	*BMJ Open*
Manuscript ID	bmjopen-2019-031210.R1
Article Type:	Protocol
Date Submitted by the Author:	16-May-2019
Complete List of Authors:	Richards, James; Cambridge University, Department of Surgery; Addenbrooke's Hospital, Department of Surgery<br>Hossain, Mohammed; Addenbrooke's Hospital, Department of Surgery<br>Summers, Dominic ; Cambridge University, Department of Surgery; Addenbrooke's Hospital, Department of Surgery<br>Slater, Matthew; Addenbrooke's Hospital<br>Bartlett, Matthew; Royal Free London NHS Foundation Trust<br>Kosmoliaptsis, Vasilis; Cambridge University, Department of Surgery; Addenbrooke's Hospital, Department of Surgery<br>Wilson, Edward; University of Cambridge, Cambridge Centre for Health Services Research<br>Lagaac, Regin; Addenbrooke's Hospital<br>Sidders, Anna; NHSBT Clinical Trials Unit<br>Foley, Claire; NHSBT Clinical Trials Unit<br>Laing, Emma; NHSBT Clinical Trials Unit<br>Hopkins, Valerie; NHSBT Clinical Trials Unit<br>Fitzpatrick-Creamer, Chloe; NHSBT Clinical Trials Unit<br>Hudson, Cara; NHSBT Clinical Trials Unit<br>Thomas, Helen; NHSBT Clinical Trials Unit<br>Turner, Sam; North Bristol NHS Trust<br>Tambyraja, Andrew; Royal Infirmary of Edinburgh<br>somalanka, Subash; Epsom and St Helier University Hospitals NHS Trust<br>Hunter, James; University Hospitals Coventry and Warwickshire NHS Trust<br>Dutta, Sam; Nottingham University Hospitals NHS Trust<br>Lawman, Sarah; Brighton and Sussex University Hospitals NHS Trust<br>Salter, Tracey; Frimley Health NHS Foundation Trust; Epsom and St Helier University Hospitals NHS Trust<br>Aslam, Mohammed; Imperial College London Department of Surgery and Cancer, Department of Vascular Surgery<br>Bagul, Atul; University Hospitals of Leicester NHS Trust<br>Sivaprakasam, Rajesh; Barts Health NHS Trust<br>Smith, George; Hull University Teaching Hospitals NHS Trust<br>Moinuddin, Zia; Manchester University NHS Foundation Trust<br>Knight, Simon; University of Oxford,<br>Gibbs, Paul; Portsmouth Hospitals NHS Trust, Renal Transplant Department, Portsmouth Hospitals NHS Trust<br>Motallebzadeh, Reza; Royal Free London NHS Foundation Trust<br>Barnett, Nicholas; Guy's and Saint Thomas' Hospitals NHS Trust

	Pettigrew, Gavin ; Cambridge University, Department of Surgery; Addenbrooke's Hospital, Department of Surgery
**Primary Subject Heading**:	Surgery
Secondary Subject Heading:	Evidence based practice, Radiology and imaging, Renal medicine, Surgery
Keywords:	End stage renal failure < NEPHROLOGY, Dialysis < NEPHROLOGY, SURGERY, Ultrasound < RADIOLOGY & IMAGING

SCHOLARONE™
Manuscripts

A study protocol for the multi-centre observational study: Surveillance Of arterioveNous fistulAe using ultRasound (SONAR) trial in haemodialysis patients

James Richards ^[1,2], Mohammed Hossain ^[1], Dominic Summers [1,2], Matthew Slater [1], Matthew Bartlett [3], Vasilis Kosmoliaptsis [1,2], Edward Wilson [2], Regin Lagaac [1], Anna Sidders [4], Claire Foley [4], Emma Laing [4], Valerie Hopkins [4], Chloe Fitzpatrick-Creamer [4], Cara Hudson [4], Helen Thomas [4], Sam Turner [5], Andrew Tambyraja [6], Subash Somalanka [7], James Hunter [8], Sam Dutta [9], Sarah Lawman [10], Tracey Salter [7,11], Mohammed Aslam [12], Atul Bagul [13], Rajesh Sivaprakasam [14], George Smith [15], Zia Moinuddin [16], Simon Knight [17], Paul Gibbs [18], Reza Motallebzadeh [3], Nicholas Barnett [19], Gavin Pettigrew *[1,2].

On behalf of the SONAR trial group.

[1] Addenbrooke's Hospital, Hill Road, Cambridge
[2] University of Cambridge, Hill Road, Cambridge
[3] Royal Free London NHS Foundation Trust
[4] NHS Blood and Transplant Clinical Trials Unit
[5] North Bristol NHS Trust
[6] Royal Infirmary of Edinburgh, NHS Lothian
[7] Epsom and St Helier University Hospitals NHS Trust
[8] University Hospital Coventry and Warwickshire NHS Trust
[9] Nottingham University Hospitals NHS Trust
[10] Brighton & Sussex University Hospitals NHS Trust
[11] Frimley Health NHS Foundation Trust
[12] Imperial College Healthcare NHS Trust
[13] University Hospitals of Leicester NHS Trust
[14] Bart's Health NHS Trust
[15] Hull University Teaching Hospitals NHS Trust
[16] Manchester University NHS Foundation Trust
[17] Oxford University Hospitals NHS Foundation Trust
[18] Portsmouth Hospitals NHS Trust)
[19] Guy's & St Thomas' NHS Foundation Trust

* Corresponding author & Chief Investigator: Mr Gavin Pettigrew, Department of Surgery, Box 202, Level E9, Addenbrooke's Hospital, Cambridge Biomedical Campus, Hills Road, Cambridge, CB2 0QQ, UK. Email: gjp25@cam.ac.uk. Tel: 01223 336976. Fax 01223 762523.

^ Joint first authors

Abstract

Introduction

Arteriovenous fistulas (AVFs) are considered the best and safest modality for providing haemodialysis in patients with End-Stage Renal Disease. Only 20% of UK centres achieve the recommended 80% target for achieving dialysis of the prevalent dialysis population via

For peer review only

permanent access (as opposed to a central venous catheter). This is partly due to the relatively poor maturation rate of newly created fistulas, with as many as 50% of fistulas failing to mature.

The SONAR study will examine whether a protocolised programme of Doppler ultrasound (US) surveillance can identify, early after creation, potentially correctable problems in those AVFs that subsequently fail to mature.

Methods and Analysis
This is a multi-centre observational study that will assess newly created AVFs by Doppler US performed at 2, 4, 6 and 10 weeks after creation. The primary outcome measure will be primary fistula patency at week 10. Secondary outcome measures include: successful use of the fistula; clinical suitability for dialysis; creation of new fistula or radiological salvage; fistula thrombosis; secondary fistula patency rate; and patient acceptability.

Ethics and dissemination
The study has been approved by the Cambridgeshire and Hertfordshire Research Ethics Committee and by the Health Research Authority (REC 18/EE/0234). The results generated from this work will be published as open access, within three years of trial commencement. We will also present our findings at key national / international renal meetings, as well as support volunteers at renal patient groups to disseminate the trial outcome.

Trial Registration number
This study is registered with the ISRCTN Clinical Trials Register (ISRCTN36033877).

Manuscript Word Count: 3615

For peer review only

Strengths and limitations of this study

- It is a prospective observational multi-centre study that examines whether a programme of Doppler Ultrasound (US) surveillance can identify those newly created arteriovenous fistulas (AVFs) that are unlikely to mature.
- The demonstration that US surveillance can successfully identify newly-created AVFs that are unlikely to mature will prompt a second-phase prospective randomised study, in which 'at-risks' fistulas are randomised to continued observation (control arm) or attempted surgical / radiological salvage.
- The second-phase study will use the same centres and trial expertise as assembled for the initial observational study and would represent the largest vascular access study yet performed, with ~1200 participants.
- The main limitation of this study is that the primary end-point uses a surrogate marker of fistula patency (based on US flow characteristics), rather than an independent and functionally-relevant endpoint (successful use of the fistula for dialysis).

Keywords
Arteriovenous Fistula
Vascular Access Surgery
Doppler Ultrasound
Haemodialysis
End-stage Kidney Disease

For peer review only

Introduction

The kidneys provide a number of key functions, including the excretion of excess fluid and harmful toxins. In patients with end-stage renal disease (ESRD), the excessive build-up of toxins and fluid would be fatal if left untreated. Consequently, patients with ESRD require renal replacement therapy (RRT), in the form of either kidney transplantation or dialysis. About 70% of patients requiring dialysis opt for haemodialysis (equating to about 20,000 patients a year in the UK) [1].

Arteriovenous fistulas (AVFs) are considered the best modality for providing haemodialysis in patients with ESRD, because haemodialysis via a central venous catheter (CVC) is associated with increased incidence of bloodstream infection, hospitalisation and cost [2-8]. Patient mortality for patients dialysing via a CVC is about 40% higher than for patients dialysing via an AVF [9].

Only 20% of UK dialysis centres currently achieve the 80% Renal Association target for dialysis of their prevalent population via definitive access (rather than a CVC) [1 10]. The reasons why such a small proportion achieve dialysis via an AVF are multifactorial, but the relatively poor maturation rate, with as many as 50% of fistulas failing to mature, undoubtedly contributes. Although this may reflect sub-optimal arterial inflow that is difficult to rectify, stenosis due to venous intimal hyperplasia also contributes, and is potentially correctable by either radiological or surgical intervention. If so, the improved assisted primary fistula patency is likely to increase AVF usage substantially, as well as saving money by avoiding the need to create a further AVF or to dialyse via a CVC. Increased AVF usage may result in improved patient survival by avoiding CVC-related complications, and by preserving precious venous 'capital' for future fistula formation.

The literature relating to ultrasound (US) surveillance of AVFs is conflicting. This may reflect variations in the type of surveillance adopted, the type of fistula under surveillance and the precise US scanning method. US can reliably identify fistulas that have successfully matured [11 12], and although the precise ultrasonic characteristics that constitute a mature fistula continue to be debated [13], adoption of a surrogate US definition of maturity avoids exclusion of pre-dialysis patients from vascular access trials.

Few studies have attempted to use ultrasound to characterise early maturation, immediately after fistula creation, but those that do suggest that successful fistula maturation is associated with rapid increase in fistula blood flow, even by the first day after formation [14 15]. Fistula vein diameter may also increase rapidly [15]. Thus, assessment at these earlier time points may be predictive of later patency. In a study of 153 patients, Itoga et al. performed early duplex US on newly-formed fistulas (4-8 weeks after creation) [16]. A flow limiting stenosis was detected in 40% of patients, of whom 81% underwent subsequent radiological intervention. Assisted primary patency of the fistulas in this group (compared to the cohort without detectable US abnormality) was 83% vs 96% at six months and 64% vs 89% at one year. There was no control cohort (patients who did not undergo routine surveillance), but the assisted patency reported for the entire study population would appear to be better than generally reported following fistula creation. One randomised study has evaluated routine early US surveillance (2, 4 and 8 weeks after fistula creation,

For peer review only

150 patients), and reported a 13.6% fistula failure/non-maturation rate in the surveillance group, compared to 25.4% in the control group in whom US was performed on the basis of a perceived clinical indication [17]. This difference did not reach statistical significance, but notably, the study was powered for a 20% difference in maturation. Our proposed study is powered for a 10% difference, with a corresponding increase in numbers of enrolled patients.

Rationale for Study
Only 20% of UK dialysis centres manage to meet the Renal Association guidelines to dialyse their prevalent population via an AVF. Numerous factors contribute to this, but the high early failure rate, with as many as 50% of nascent fistulas failing to mature, is undoubtedly a major factor.

At present, the paucity of evidence suggesting surveillance increases rates of dialysis via the fistula alongside the significant cost and required resources, leads to significant heterogeneity in practice across the UK and internationally. This study aims to fill this gap in knowledge by determining whether US surveillance of newly-formed AVFs can be used to identify failing fistulas.

We aim to demonstrate whether a structured programme of Doppler US surveillance will accurately predict structural abnormalities (such as venous intimal hyperplasia) that lead to failure of a newly-created fistula to adequately develop and provide effective access for dialysis. We hypothesise that these detected structural abnormalities would be amenable to correction by interventional radiology or surgery, leading to improved assisted fistula patency. This would likely increase AVF usage substantially, as well as potentially save money by avoiding the need to create a further AVF or to dialyse via a CVC. Increased AVF usage may result in improved patient survival by avoiding CVC-related complications, and by preserving precious venous 'capital' for future fistula formation.

When after fistula creation should US surveillance be performed?
Although one year unassisted fistula patency (primary) is approximately 55%, about 60% of these failures will occur within the first three months after creation [18 19], and it is likely that failures in the first year relate to individuals whose fistula had never developed optimally [20 21]. This study therefore assesses US surveillance of the fistula during its maturation phase, immediately after creation.

We feel that for a trial to demonstrate that US surveillance improves patency rates for newly created fistulas, two conditions must be met:

1. Ultrasound can effectively distinguish those newly-formed fistulas that are unlikely to mature.
2. Salvage interventions performed on those 'at-risk' fistulas are effective.

Study Design
These conditions will be addressed in two phases:
Phase 1: A prospective observational cohort study to firstly determine whether US surveillance can reliably predict fistula failure.

For peer review only

Phase 2: A prospective randomised trial that examines one-year fistula patency and compares US-directed fistula salvage against standard clinical assessment (no ultrasound surveillance).

This study protocol covers only phase 1 of the study. We will only move on to phase 2 if US is found to be effective at identifying 'at-risk' AVFs: this will be covered by a separate protocol. The outputs of phases 1 and 2 will form the primary inputs into a decision model predicting the incremental cost effectiveness of US surveillance versus standard clinical assessment.

Phase 1 will recruit patients who are either pre-dialysis or already established on haemodialysis via a central venous catheter (see Figure 1). Consenting patients will undergo serial US scanning at weeks 2, 4, 6 and 10 after fistula formation in addition to standard care (such as regular clinical assessment) as per local centre policy. The US findings will be blinded, i.e. will not be relayed to the participant or to the participant's clinical team.

Potential benefits and risks of study

The only direct benefit to the participating patient is the detection of fistula thrombosis at an earlier juncture than would normally occur with routine clinic follow up. We anticipate, however, that the incentive to participate in a trial that clarifies the role of ultrasound surveillance of newly-formed AVFs will enable recruitment rates to be met.

Doppler US is a low risk form of imaging, as it does not use ionizing radiation and as such this observational study poses very low risk to participants. Acoustic power output and duration of exposure to ultrasound should not exceed those of a typical diagnostic examination, with exposure kept as low as reasonably achievable [22]. While no patient injury has been recorded from non-contrast enhanced US at diagnostic levels, bioeffects including significant heating and cavitation have been demonstrated at higher intensities.

Methods and Analysis

Study Hypothesis

Doppler US surveillance can reliably predict failing nascent AV fistulas by identifying potentially correctable anatomical defects.

Intervention

This study will be performed in the UK across 15 participating centres starting 1st August 2018 and with the aim to complete recruitment by 31st October 2019. Consenting participants will be observed for 10 weeks following creation of their AV fistula and will undergo Doppler US scans during weeks 2, 4, 6 and 10. Routine clinical examination will be undertaken as per local policy with a final clinical examination at week 10 to evaluate the fistula.

Doppler Ultrasound Surveillance

Duplex Doppler US machines used for the study must have B-mode imaging frequencies of at least 7MHz. The machines will have both high (>12MHz), midrange (>7MHz) and curvilinear (approximately 3MHz) probes. Scans will be carried out by vascular scientists, surgeons, sonographers and other personnel trained in arteriovenous fistula surveillance by

For peer review only

Doppler US; training days have been provided to ensure a standardised approach. The parameters measured for the study will be recorded on an agreed study proforma and include: brachial artery (or subclavian/axillary artery in the presence of a high bifurcation) flow volume and resistance index, outflow vein diameter, anastomotic diameter, AVF depth, presence of a flow limiting stenosis and fistula thrombosis.

Definitions used within study

Primary patency:

The interval between access creation to thrombosis event or first surgical/radiological intervention.

Secondary patency

The interval between access creation to abandonment of the access including all radiological and surgical salvage procedures in between.

Clinical maturation

Suitability to cannulate based on clinical examination

Functional maturation

Ability for the access to achieve adequate dialysis

Inclusion Criteria

1. Aged 16 years or older
2. Can provide fully informed consent
3. Have end-stage renal disease and require haemodialysis or are likely to do so imminently
4. Are due creation of a wrist or elbow arteriovenous fistula, including the following types of fistula with a minimal acceptable threshold of 2 mm venous diameter (with a tourniquet applied) at the site chosen:
    - Radiocephalic
    - Ulnobasilic
    - Brachiocephalic
    - Brachiobasilic

Exclusion criteria

1. Attempted formation of proximal neo-anastomosis at the forearm cephalic and basilic venous systems following failure of a standard radiocephalic or ulnobasilic fistula.
2. Known central venous stenosis (including those who undergo simultaneous central venous angioplasty / stenting and arteriovenous fistula creation).
3. Anticipated that it will not be possible to perform serial ultrasound scanning.

For peer review only

Primary outcome measure
Primary fistula patency at week 10, according to surrogate ultrasound parameters. This is assessed using venous diameter and blood flow measurements:

- Wrist fistula considered to be patent if there is a minimum venous diameter of 4 mm with a blood flow measurement of >400 mls/min
- Elbow fistula, considered to be patent if there is a minimum venous fistula diameter of 5 mm, with a blood flow measurement of >500 mls/min

Secondary outcome measures
1. For those patients established on dialysis, successful use of the fistula, determined by its use for dialysis on 3 separate occasions during the 10 weeks after the arteriovenous fistula (AVF) surgical creation.
2. Clinical suitability for dialysis based on examination alone according to local practice, assessed 10 weeks after AVF surgical creation.
3. Formation of a new fistula (including fashioning of proximal neoanastomosis) or radiological salvage procedure, measured by collecting the number and type of these interventions during the 10 weeks after AVF surgical creation.
4. Fistula thrombosis rate: the number of fistulae that thrombose during the 10 weeks after surgical creation.
5. Secondary fistula patency rate: measured by the time interval (in days) between AVF creation until abandonment of the AVF, including all radiological and surgical salvage procedures in between, during the 10 weeks after AVF surgical creation.
6. Patient acceptability based on the proportion of patients that complete their study ultrasound scans 10 weeks after AVF surgical creation.

Blinding
Both the patient and the treating clinical team will be blinded to the results of the Doppler US.

The only acceptable reasons for unblinding are:

1. The participating centre's local standard of care requires a scan, or a clinical need for a scan is identified. In which case the centre will have access to study scan data for that timepoint (but not the other study scans) to avoid unnecessary additional scans being scheduled.

Or:

2. During a trial scan, the AVF is seen to be thrombosed, in which case this information would be shared with the clinical care team to enable appropriate care to continue. In such cases, no further study scans would be required. Clinical outcome data will still be collected at week 10.

Sample size
We have estimated that 20% of fistulas fail early and that early ultrasound has a positive predictive value (number of true positives/number of predicted positives, PPV) of 72% for predicting non-maturation. To estimate this with ±10% precision (i.e. the 95% confidence interval is from 62% to 82%), 78 predicted failures are required. We estimate that US

For peer review only

predicts failure in 25% of fistulas meaning 312 fistulas are required in the study. Allowing for 10% dropout, 347 fistulas will be recruited. In order to predict primary fistula patency for the phase 2 trial (not part of this protocol), we anticipate that two models will be required and therefore we will have two PPVs- one for wrist fistulas and one for elbow fistulas. Assuming a ratio of 50:50 for wrist to elbow fistulas, the precision confidence interval will be from 55.3% to 85.2%.

Analysis Plan

All analyses will be performed according to intention to treat. The analysis population will include all participants enrolled into the study, including those whose fistula failed within the first two weeks post-surgery and those enrolled in error.

Interim Monitoring and Analyses

The feasibility of recruitment will be assessed based on data between month 3 and month 9 of the recruitment period. Stop-go criteria for expanding to complete the full first phase trial will be used. The trial will be stopped if fewer than 80 patients have been recruited, additional centres will be recruited if less than 120 patients have been recruited, otherwise the trial will continue as planned.

Analysis of primary and secondary outcomes

The primary fistula patency rate at week 10 will be calculated alongside an exact 95% confidence interval based on all participants enrolled. It will also be calculated based on participants whose fistulas did not fail early. Patency will be assessed using the results from each ultrasound scan up to week 10.

The secondary outcomes will be presented using descriptive summary statistics.

Mixed multivariable logistic regression will be used to model primary fistula patency by 10 weeks, which will then be used as a risk-score calculator in the phase 2 trial. Two models will be built, one for wrist fistulas and one for elbow fistulas. Measurements taken from the first scan performed at week 2 will be considered as parameters in the model initially and further parameters from either the second scan or the third scan will then be considered. The choice of second or third scan will be based on the best fitting parameters, as assessed by significance level. A random effect for the participant will be included in the models to account for multiple scan data per participant. The aim will be to build parsimonious models, which contain the minimum number of measurements required to effectively predict primary fistula patency. A receiver operating characteristic (ROC) curve analysis of the developed risk scores will be used to assist decisions regarding an appropriate cut-off for an indicator for when intervention is required for both wrist and elbow fistulas. The PPV will be calculated alongside an exact 95% confidence interval for the chosen risk-score cut-off.

It is anticipated that some participants will not attend all scans and therefore some scan data will be missing. Levels of missing data will be summarised for each of the scan time points and will be considered when choosing which scan results to use in the final model. Any missing primary and secondary outcome data will not be imputed. Missing data for

For peer review only

parameters in the modelling of primary fistula patency will be imputed using multiple imputation if the level of missing data is greater than 10%.

Data management

Study specific data, which is non-identifiable, will be collected at each site on the Case Report Form (CRF). Each participant will have a unique ID allocated to them which will be recorded on the CRF for reporting purposes. Only study sites will have access to the identifiable information to maintain participant confidentiality. CRF data will be submitted to NHSBT Clinical Trials Unit (CTU) at pre-specified intervals and logged into the regulatory compliant, secure MACRO™ database. Only authorised personnel at NHSBT CTU will have password protected access to the study database.

CRFs, clinical notes and administrative documentation will be kept in a secure location (e.g. locked filing cabinets in a room with restricted access) and held for 10 years after the end of the study. During this period, all data will be accessible to the competent authorities and the Sponsor with suitable notice.

Study Closure

The study will be closed after the last 10-week appointment of the last recruited patient is completed and all data have been received.

Patient and Public Involvement

We discussed trial design and ethical considerations with a number of our haemodialysis patients, as well as focus group consisting of 12 lay individuals. As well as considering the trial as a whole, they specifically considered two-stage design of the trial, the time commitment for participants and also supported the blinding of results from the patient's medical team.

Ethics and dissemination

Ethical and Regulatory Issues

The Cambridgeshire and Hertfordshire Research Ethics Committee has approved this study (18/EE/0234). SONAR complies with the Declaration of Helsinki and will be conducted in compliance with the approved protocol, the principles of Good Clinical Practice (GCP), the UK Data Protection Act, the General Data Protection Regulation and the UK Policy Framework for Health and Social Care Research.

Ethical Considerations of SONAR study

As the study is observational with a small likelihood of direct benefit to participants, their involvement will be mainly altruistic. This will be made clear during discussions with potential participants and is clearly stated in the patient information sheet

Consent

The rights of the patient to refuse to participate in the study without giving a reason will be respected. After the participant has entered into the study, the clinician remains free to treat the patient according to best standards of care, irrespective of their involvement in the study. The participant will remain within the study for the purposes of follow up and for

For peer review only

data analysis. Similarly, the participant will remain free to change their mind to participate at any time without giving a reason and without prejudicing his/her further treatment.

Publication and dissemination plan

The results generated from this work will be published in open access, peer reviewed papers and presented at national/international conferences.

Access to data

The datasets generated during and/or analysed during the current study will be available upon request from Mr Gavin Pettigrew (gjp25@cam.ac.uk). Participants have consented to non-identifiable results to be publicly available. No identifiable data will be shared.

Discussion

SONAR will be one of the largest observational vascular access studies yet performed. If Doppler US accurately identifies anatomically correctable lesions that are preventing maturation ("salvageable fistula"), this will be used to power and justify a second phase interventional study that addresses whether timely surgical and/or radiological intervention improves fistula patency rates. Conversely, if the SONAR study does not identify a role for routine Doppler US surveillance of newly-formed arteriovenous fistulas, this would have immediate relevance for studies in other countries that are similarly using Doppler US to assess fistula maturation [21 23 24].

Authors' contributions

JR, MH, MS, DS, MB, VK, EW, RL, AS, CF, EL, VH, CFC, CH, HT, ST, AT, SS, JH, SD, SL, TS, MA, AB, RS, GS, ZM, SK, PG, RM, NB & GJP contributed to the development and implementation of this protocol and have approved this manuscript. All members of the Trial Group were involved in the development of the study.

SONAR TRIAL Group

Anna Sidders, Cara Hudson, Claire Foley, Valerie Hopkins, Emma Laing, Chloe Fitzpatrick-Creamer, Helen Thomas, Alison Deary (NHS Blood and Transplant Clinical Trials Unit)

Gavin Pettigrew, James Richards, Mohammed Hossain, Dominic Summers, Matthew Slater, Regin Lagaac, Veena Surendrakumar, Tobi Ayorinde, Igor Chipurovski, Subhankar Paul (Cambridge University Hospital)

Simon Knight, Klaus Bond, Elizabeth Hardy, Joanne Widdup, Rachael Potter, Elisabeth Pugh, Karen Parsons, Kathryn Lafferty (Oxford University Hospitals NHS Foundation Trust)

Rajesh Sivaprakasam, Kate Crawford, Amy Bolsworth, Naavalah Ngwa-Ndifor (Bart's Health NHS Trust)

Reza Motallebzadeh, Matthew Bartlett, Rani Badhan (Royal Free London NHS Foundation Trust)

Zia Moinuddin, Helena Edlin, Anna Jerram, Jessica Lai, Joyce Banda, Janet Bendle (Manchester University NHS Foundation Trust)

For peer review only

Sam Turner, Maria Morgan, William Owen, Sue Dawson, Simon Daniel, Karen Allsop, (North Bristol NHS Trust)

Andrew Tambyraja, Sarah-Jane Carmichael, Tom Eadie, Rona Lochiel, Midel Lena (Royal Infirmary of Edinburgh, NHS Lothian)

Nicholas Barnett, Soundrie Padayachee, Philip Eldridge, May Rabuya (Guy's & St Thomas' NHS Foundation Trust)

Subash Somalanka, Jashree Patel, Abbas Ghazanfar, Judy van Selm, Caroline Bodneck, Martia Augustin, Kwame Ansu, Nalin Khosla, Kashif Burney, Karen Dear (Epsom and St Helier University Hospitals NHS Trust)

James Hunter, Carl Tiivas, Samantha Hyndman, Maria Truslove (University Hospital Coventry and Warwickshire NHS Trust)

Sam Dutta, Andrew Beech, Sarah Brand (Nottingham University Hospitals NHS Trust)

Sarah Lawman, Darren Cheal, Mel Smith, Kate Trivedi (Brighton & Sussex University Hospitals NHS Trust)

Tracey Salter, Adnan Bajwa, John Kerr, Ana Fleet, Lianne Chapman, Sarah Gee, Thanuja Weerasinghe (Frimley Health NHS Foundation Trust)

George Smith, Paris Cai, Judith Long, Tracey Rowe (Hull University Teaching Hospitals NHS Trust)

Mohammed Aslam, Jeremy Crane (Imperial College Healthcare NHS Trust)

Atul Bagul, Mary Quashie-Akponeware (University Hospitals of Leicester NHS Trust)

Paul Gibbs (Portsmouth Hospitals NHS Trust)

Funding Statement

This study is funded by National Institute for Health Research (NIHR) Health Technology Assessment Board (grant reference 17/27/11). The views expressed are those of the author(s) and not necessarily those of the NIHR or the Department of Health and Social Care. JR and DS are funded by NIHR Academic Clinical Lectureships.

Competing interests

None declared

For peer review only

References

1. Caskey F, Cullen R. UK Renal Registry 18th Annual Report: Introduction. *Nephron* 2016;132 Suppl 1:1-8. doi: 10.1159/000444814 [published Online First: 2016/04/19]
2. Taylor G, Gravel D, Johnston L, et al. Incidence of bloodstream infection in multicenter inception cohorts of hemodialysis patients. *Am J Infect Control* 2004;32(3):155-60. doi: 10.1016/j.ajic.2003.05.007 [published Online First: 2004/05/22]
3. D'Agata EM, Mount DB, Thayer V, et al. Hospital-acquired infections among chronic hemodialysis patients. *Am J Kidney Dis* 2000;35(6):1083-8. [published Online First: 2000/06/10]
4. Marr KA, Kong L, Fowler VG, et al. Incidence and outcome of Staphylococcus aureus bacteremia in hemodialysis patients. *Kidney Int* 1998;54(5):1684-9. doi: 10.1046/j.1523-1755.1998.00134.x [published Online First: 1998/12/09]
5. Rodriguez-Aranda A, Alcazar JM, Sanz F, et al. Endoluminal colonization as a risk factor for coagulase-negative staphylococcal catheter-related bloodstream infections in haemodialysis patients. *Nephrol Dial Transplant* 2011;26(3):948-55. doi: 10.1093/ndt/gfq481 [published Online First: 2010/08/13]
6. Rosenbaum D, MacRae JM, Djurdjev O, et al. Surveillance cultures of tunneled cuffed catheter exit sites in chronic hemodialysis patients are of no benefit. *Hemodial Int* 2006;10(4):365-70. doi: 10.1111/j.1542-4758.2006.00131.x [published Online First: 2006/10/04]
7. McCann M, Moore ZE. Interventions for preventing infectious complications in haemodialysis patients with central venous catheters. *Cochrane Database Syst Rev* 2010(1):Cd006894. doi: 10.1002/14651858.CD006894.pub2 [published Online First: 2010/01/22]
8. Engemann JJ, Friedman JY, Reed SD, et al. Clinical outcomes and costs due to Staphylococcus aureus bacteremia among patients receiving long-term hemodialysis. *Infect Control Hosp Epidemiol* 2005;26(6):534-9. doi: 10.1086/502580 [published Online First: 2005/07/16]
9. Astor BC, Eustace JA, Powe NR, et al. Type of vascular access and survival among incident hemodialysis patients: the Choices for Healthy Outcomes in Caring for ESRD (CHOICE) Study. *J Am Soc Nephrol* 2005;16(5):1449-55. doi: 10.1681/asn.2004090748 [published Online First: 2005/03/25]
10. Association TR. Vascular Access Guidelines https://renal.org/wp-content/uploads/2017/06/vascular-access.pdf 2015 [2.
11. Ives CL, Akoh JA, George J, et al. Pre-operative vessel mapping and early post-operative surveillance duplex scanning of arteriovenous fistulae. *J Vasc Access* 2009;10(1):37-42.
12. Hindle P, Ritchie J, Griffiths G, et al. Use of early scanning in fistula formation for vascular access. *The Journal of Vascular Access* 2011;12(2):127-29. doi: 10.5301/jva.2010.6072
13. Beathard GA, Lok CE, Glickman MH, et al. Definitions and End Points for Interventional Studies for Arteriovenous Dialysis Access. *Clin J Am Soc Nephrol* 2018;13(3):501-12. doi: 10.2215/cjn.11531116 [published Online First: 2017/07/22]
14. Saucy F, Haesler E, Haller C, et al. Is intra-operative blood flow predictive for early failure of radiocephalic arteriovenous fistula? *Nephrol Dial Transplant* 2010;25(3):862-7. doi: 10.1093/ndt/gfp577

For peer review only

15. Robbin ML, Greene T, Cheung AK, et al. Arteriovenous Fistula Development in the First 6 Weeks after Creation. *Radiology* 2016;279(2):620-9. doi: 10.1148/radiol.2015150385
16. Itoga NK, Ullery BW, Tran K, et al. Use of a proactive duplex ultrasound protocol for hemodialysis access. *J Vasc Surg* 2016;64(4):1042-49 e1. doi: 10.1016/j.jvs.2016.03.442
17. Han A, Min SK, Kim MS, et al. A Prospective, Randomized Trial of Routine Duplex Ultrasound Surveillance on Arteriovenous Fistula Maturation. *Clin J Am Soc Nephrol* 2016;11(10):1817-24. doi: 10.2215/CJN.00620116
18. Wells AC, Fernando B, Butler A, et al. Selective use of ultrasonographic vascular mapping in the assessment of patients before haemodialysis access surgery. *Br J Surg* 2005;92(11):1439-43. doi: 10.1002/bjs.5151 [published Online First: 2005/09/28]
19. Huijbregts HJ, Bots ML, Wittens CH, et al. Hemodialysis arteriovenous fistula patency revisited: results of a prospective, multicenter initiative. *Clin J Am Soc Nephrol* 2008;3(3):714-9. doi: 10.2215/CJN.02950707
20. Robbin ML, Chamberlain NE, Lockhart ME, et al. Hemodialysis arteriovenous fistula maturity: US evaluation. *Radiology* 2002;225(1):59-64. doi: 10.1148/radiol.2251011367 [published Online First: 2002/10/02]
21. Singh P, Robbin ML, Lockhart ME, et al. Clinically immature arteriovenous hemodialysis fistulas: effect of US on salvage. *Radiology* 2008;246(1):299-305. doi: 10.1148/radiol.2463061942 [published Online First: 2007/11/10]
22. American Institute of Ultrasound in Medicine Official Statement on Safety in Training and Research, 2012. 2012
23. Robbin ML, Greene T, Allon M, et al. Prediction of Arteriovenous Fistula Clinical Maturation from Postoperative Ultrasound Measurements: Findings from the Hemodialysis Fistula Maturation Study. *J Am Soc Nephrol* 2018;29(11):2735-44. doi: 10.1681/asn.2017111225 [published Online First: 2018/10/13]
24. Robbin ML, Greene T, Cheung AK, et al. Arteriovenous Fistula Development in the First 6 Weeks after Creation. *Radiology* 2016;279(2):620-9. doi: 10.1148/radiol.2015150385 [published Online First: 2015/12/24]

For peer review only

FIGURE LEGEND

Figure 1: SONAR study flow chart
Flow chart of patient journey from screening and enrolment through to final study scan and clinical assessment within the SONAR trial.

For peer review only

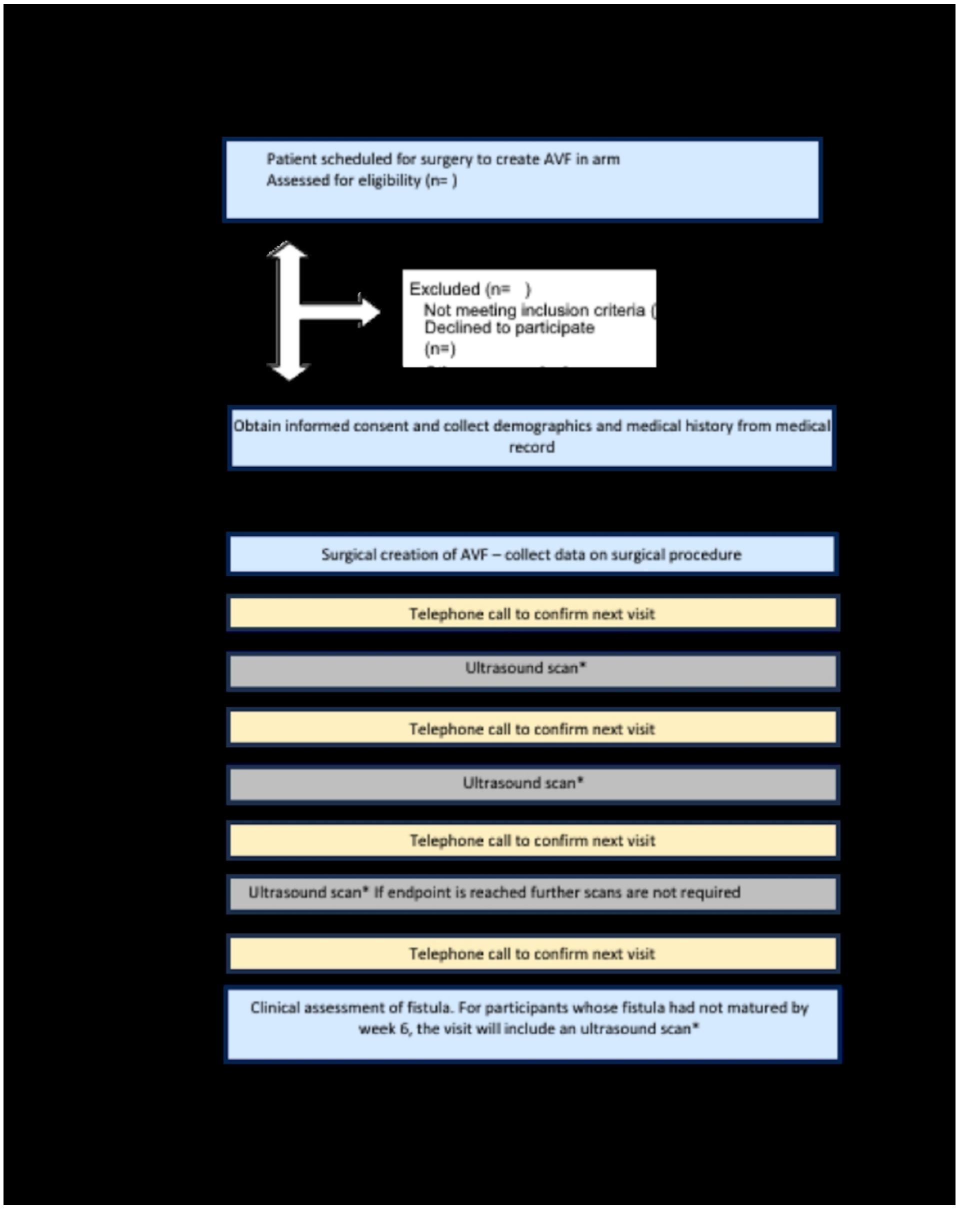

Figure 1: SONAR study flow chart
Flow chart of patient journey from screening and enrolment through to final study scan and clinical assessment within the SONAR trial.

119x150mm (300 x 300 DPI)
