# Supplementary material for: Surveillance arterioveNous fistulAs using ultRasound (SONAR) trial in haemodialysis patients: a study protocol for a multicentre observational study
Source: BMJ Open. 2019 Jul 23;9(7):e031210. doi: 10.1136/bmjopen-2019-031210 (PMC6661628; doi:10.1136/bmjopen-2019-031210)
Supplement: Reviewer comments [file bmjopen-2019-031210.reviewer_comments.pdf]

PEER REVIEW HISTORY

BMJ Open publishes all reviews undertaken for accepted manuscripts. Reviewers are asked to complete a checklist review form (**http://bmjopen.bmj.com/site/about/resources/checklist.pdf**) and are provided with free text boxes to elaborate on their assessment. These free text comments are reproduced below.

ARTICLE DETAILS

**TITLE (PROVISIONAL)**	A study protocol for the multi-centre observational study: Surveillance Of arterioveNous fistulAe using ultRasound (SONAR) trial in haemodialysis patients
**AUTHORS**	Richards, James; Hossain, Mohammed; Summers, Dominic; Slater, Matthew; Bartlett, Matthew; Kosmoliaptsis, Vasilis; Wilson, Edward; Lagaac, Regin; Sidders, Anna; Foley, Claire; Laing, Emma; Hopkins, Valerie; Fitzpatrick-Creamer, Chloe; Hudson, Cara; Thomas, Helen; Turner, Sam; Tambyraja, Andrew; somalanka, Subash; Hunter, James; Dutta, Sam; Lawman, Sarah; Salter, Tracey; Aslam, Mohammed; Bagul, Atul; Sivaprakasam, Rajesh; Smith, George; Moinuddin, Zia; Knight, Simon; Gibbs, Paul; Motallebzadeh, Reza; Barnett, Nicholas; Pettigrew, Gavin

This article was not externally reviewed at BMJ Open. Protocols that have been independently assessed prior to submission to BMJ Open are usually fast-tracked to publication on the grounds that further substantial changes will not be possible. This independent assessment will usually be external, independent review for both a substantial grant award from a non-commercial or government funder and ethics approval.
